# Nosocomial Pneumonia in the Era of Multidrug-Resistance: Updates in Diagnosis and Management

**DOI:** 10.3390/microorganisms9030534

**Published:** 2021-03-05

**Authors:** Elena Xu, David Pérez-Torres, Paraskevi C. Fragkou, Jean-Ralph Zahar, Despoina Koulenti

**Affiliations:** 1 Burns, Trauma and Critical Care Research Centre, University of Queensland Centre for Clinical Research, Faculty of Medicine, The University of Queensland, Brisbane, QLD 4029, Australia; elena.xu@uq.net.au; 2 Servicio de Medicina Intensiva, Hospital Universitario Río Hortega, 47012 Valladolid, Spain; dperezt@saludcastillayleon.es; 3 Fourth Department of Internal Medicine, Attikon University Hospital, 12462 Athens, Greece; pcfragkou@med.uoa.gr; 4 Microbiology Department, Infection Control Unit, Hospital Avicenne, 93000 Bobigny, France; jrzahar@gmail.com; 5 Second Critical Care Department, Attikon University Hospital, 12462 Athens, Greece

**Keywords:** nosocomial pneumonia, hospital-acquired pneumonia, ventilator-associated pneumonia, lung ultrasound, low-radiation CT, rapid microbiological diagnosis, syndromic multiplex PCR panels, novel antibiotics

## Abstract

Nosocomial pneumonia (NP), including hospital-acquired pneumonia in non-intubated patients and ventilator-associated pneumonia, is one of the most frequent hospital-acquired infections, especially in the intensive care unit. NP has a significant impact on morbidity, mortality and health care costs, especially when the implicated pathogens are multidrug-resistant ones. This narrative review aims to critically review what is new in the field of NP, specifically, diagnosis and antibiotic treatment. Regarding novel imaging modalities, the current role of lung ultrasound and low radiation computed tomography are discussed, while regarding etiological diagnosis, recent developments in rapid microbiological confirmation, such as syndromic rapid multiplex Polymerase Chain Reaction panels are presented and compared with conventional cultures. Additionally, the volatile compounds/electronic nose, a promising diagnostic tool for the future is briefly presented. With respect to NP management, antibiotics approved for the indication of NP during the last decade are discussed, namely, ceftobiprole medocaril, telavancin, ceftolozane/tazobactam, ceftazidime/avibactam, and meropenem/vaborbactam.

## 1. Introduction

Nosocomial pneumonia (NP), comprising of hospital-acquired (HAP) and ventilator-associated pneumonia (VAP), is one of the most common nosocomial infections in the intensive care unit (ICU) and is responsible for more than half of antibiotics prescribed in the critical care settings [1,2]. HAP is defined as pneumonia, not incubating on hospital admission, developing in non-intubated patients 48 h or more after hospitalisation, while VAP is defined as pneumonia arising 48 h or more after endotracheal intubation [1,2,3,4]. VAP represents the vast majority of cases of NP in the ICU [4]. Patients with severe HAP may deteriorate further and subsequently need endotracheal intubation (ventilated HAP) [5]. Despite efforts to improve the diagnosis and management of NP, morbidity and mortality rates remain high, with mortality rates of VAP ranging from 24 to 50%, jumping to 76% if multi-drug resistant pathogens are involved [6,7,8]. Attributable mortality of VAP, on the other hand, has been debated, and reported rates vary widely and are confounded by several factors [9]. A meta-analysis of randomised VAP prevention studies reported an attributable mortality of 13%, higher in surgical patients and patients with mid-range severity scores at ICU admission [9]. Despite being considered less severe than VAP, HAP is associated with serious complications, especially when it develops in the ICU, including pleural effusions, respiratory and renal failure, septic shock and empyema in approximately 50% of the patients [2,7,10]. Both HAP and VAP lead to prolonged duration of hospitalisation and are associated with substantial healthcare costs with the mean attributable cost of VAP being USD 40,144 (95% CI USD 36,286–44,220) [11,12].

Current guidelines recommend the use of clinical criteria along with chest X-ray (CXR) to determine the need for antibiotic therapy initiation for suspected NP [2]. However owing to its varied clinical presentation, accurate clinical diagnosis of NP is difficult, especially in older patients, and there is still no diagnostic gold standard [13,14,15].

On the other hand, although a complex interplay of multiple factors contributes to the outcome of patients with NP, the accurate and timely identification of the responsible pathogen is undoubtedly important as delays in implementation of appropriate treatment may result in high mortality rates [16]. Traditional pathogen-identifying methods such as the culture-based techniques, that currently represent the gold standard in microbiological diagnosis are time consuming, requiring approximately 48–72 h before results are available [17]. This underscores an unmet need for rapid and reliable molecular tests leading to a shift from empirical to targeted antimicrobial therapy and, consequently, better clinical outcomes and less antibiotic overuse [17].

This review aims to discuss updates in the diagnosis and management of NP. Regarding diagnosis, novel imaging techniques, such as lung ultrasound (LUS) and low radiation computed tomography (LRCT) will be discussed, as well as recent developments in aetiological diagnosis, such as syndromic rapid multiplex Polymerase Chain Reaction (rm-PCR) panels. Additionally, the volatile compounds/electronic nose, a promising diagnostic tool for the future will be briefly presented. Regarding updates in NP management, antibiotics that have been approved during the last decade for the indication of NP will be discussed, namely, ceftobiprole medocaril, telavancin, ceftolozane/tazobactam, ceftazidime/avibactam and meropenem/vaborbactam [18,19].

## 2. Diagnosis of Nosocomial Pneumonia

### 2.1. Imaging Modalities

The diagnosis of HAP or VAP can be challenging for the physician, but often relies on the presence of a new or progressive radiographic infiltrate, along with clinical signs that suggest the infiltrate is of an infectious origin [1]. Accordingly, when suspected, the patient should have an imaging test. Posteroanterior and lateral CXR have been the cornerstone of the diagnosis of pneumonia for a long time. However, in the ICU, or the bedridden ward patients, portable anteroposterior CXR might show limited accuracy, therefore other imaging techniques like LRCT or LUS are gaining ground. We review the current role of CXR and the efficacy of these quite novel imaging modalities in the diagnosis of HAP or VAP.

#### 2.1.1. Chest X-ray

Lung appearance on a CXR reflects differential ventilation of alveoli, which can be affected by the presence of transudate, exudate, blood, cells or other elements (fat, proteins, water or chemicals) [20]. Occupation of alveoli by any of these elements is responsible for the classic radiological signs of pneumonia, including alveolar infiltrates with airspace opacification, air bronchogram, atelectasis, the silhouette sign or the bulging fissure sign [21].

CXR is the more widely available imaging technique and is still preferred for the diagnosis of VAP as demonstrated in a recent multicriteria decision analysis of VAP diagnosis by an expert panel [22]. However, studies have brought out important limitations of this technique because of its low accuracy, with high false positive and false negative rates. This was addressed in a systematic review by Klompas et al., where the presence of a new radiographic infiltrate minimally increased the probability of VAP (likelihood ratio 1.7, 95% CI: 1.1–2.5), relative to a histological gold standard [23]. There are at least four major reasons for this. Firstly, portable anteroposterior CXR is a common single-view projection in inpatients and almost the norm in the critically ill patient, making it difficult to optimise the patient’s position and eliminating the possibility of getting a lateral projection, thus reducing the diagnostic performance of the test [20]. Secondly, a wide range of non-infectious conditions have been described to cause NP-like radiologic patterns, which might hinder differential diagnosis: cardiogenic pulmonary oedema, pulmonary embolism, alveolar haemorrhage, pulmonary contusion, atelectasis, pleural effusion, chemical pneumonitis, cryptogenic organising pneumonia, acute eosinophilic pneumonia, vasculitis or drug reactions [15,24,25]. Poor agreement between radiologists on the interpretation of these films is common [26]. Thirdly, it is common for patients in the ward or in the ICU to have concurrent conditions which may alter the basal CXR, leading to possible overdiagnosis or misdiagnosis of NP. A classic study by Greenbaum et al. found 43% of CXR in a medical ICU showed unexpected findings or findings which caused a change in management [27]. On the other hand, a normal CXR does not exclude the presence of pulmonary disease, as demonstrated when higher resolution techniques are used [28]. Fourthly, the portable CXR dynamically changes in mechanically ventilated patients depending on the parameters set on the ventilator and mode of ventilation, leading to substantial changes in the overall diagnostic interpretation, as demonstrated by Ely et al. [29].

Diagnostic performance of NP radiographic signs was first studied by Wunderink et al., using autopsy histology as the reference standard [30]. In this study, alveolar infiltrates were highly sensitive (87.5%) but poorly specific (25.6%), whereas the presence of air bronchogram showed high sensitivity (83.3%) and modest specificity (57.8%) [30]. A recent meta-analysis by Fernando et al. on the performance of several diagnostic tests found the presence of infiltrates on CXR had a sensitivity of 88.9% (95%CI 73.9–95.8%) and a specificity of 26.1% (95%CI 15.1–41.4%) for the diagnosis of VAP, relative to the reference standard of histopathology from lung biopsy, which makes it the most sensitive but least specific of the tests assessed [31]. When combined with at least one clinical finding consistent with pneumonia (purulent secretions, fever or leukocytosis), sensitivity decreased (64.8–84.6%) and specificity remained very poor (33.6–36%), improving to 91% only when all the clinical findings were present [31]. Therefore, some multicriteria models taking CXR into account have been proposed to improve the diagnostic performance of NP, i.e., clinical pulmonary infection score (CPIS). A CPIS > 6 showed a sensitivity of 73.8% (95%CI 50.6–88.5%) and a specificity of 66.4% (95% CI 43.9–83.3%) for the diagnosis of VAP, relative to reference standard of histopathology from lung biopsy [31].

The main advantages of CXR for the diagnosis of NP are its wide availability, non-invasiveness, the possible application as a point-of-care test with portable devices, and low radiation exposure (0.1 mSv, comparable to natural background radiation for ten days) [15]. Besides diagnostic purposes, CXRs are also valuable to determine the extent of disease, to detect complications (i.e., pneumothorax, pleural effusion), to detect additional or alternative diagnoses and to guide invasive procedures [24]. Although daily routine CXR is no longer recommended in ICU patients, follow up CXR might be useful to define a differential diagnosis based on evolutive changes and to assess response to treatment. A single follow-up CXR is recommended 48 to 72 h after diagnosis, and at any time if treatment failure is suspected. A CXR improving in minutes to hours is usually associated with atelectasis, improvement in hours to days is normally related to hydrostatic oedema or haemorrhage, whereas adequately treated pneumonia tends to resolve in 4 to 6 weeks from a radiological perspective [20].

#### 2.1.2. Lung Ultrasound

Point-of-care LUS has been considered a poor performance diagnostic tool until recent times, as the energy of ultrasound is rapidly dissipated due to the air content of the healthy lung parenchyma. However, in disease conditions, the air content of the lung decreases in favour of the presence of fluid content, usually involving peripheral and subpleural areas, making it possible to examine the artefacts and patterns generated by the change of composition in the lung tissue [32].

**Figure 1:**
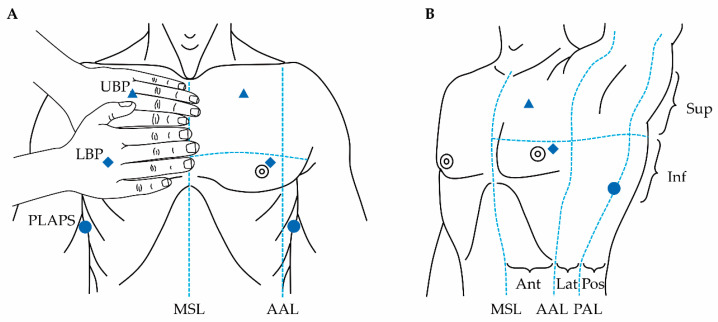
Common areas of study in LUS. (**A**) Location of the BLUE-points. Two hands of the same size as patient’s hands are used as reference, applied in a way that covers the anterior chest surface, with the upper finger positioned below the clavicle. The upper BLUE-point (UBP), represented with a triangle, is defined by the intersection of the third and fourth finger of the upper hand. The lower BLUE-point (LBP), represented with a rhombus, is defined in the middle of the lower palm. The posterolateral alveolar and/or pleural syndrome (PLAPS) point, represented with a circle, is located just above the diaphragm and behind the posterior axillary line (PAL). (**B**) Division of the hemithorax into six areas of study: three regions (anterior [Ant], lateral [Lat] and posterior [Pos]) from the front to the back, delineated by the midsternal line (MSL), the anterior (AAL) and posterior axillary lines (PAL). These areas are then subdivided into a superior (Sup) and inferior (Inf) region. BLUE points are also drawn as reference.

LUS is usually performed with a high-frequency linear probe, which allows the operator to examine the superficial areas (pleural line and derived artefacts), then switch to a low-frequency convex probe, allowing for deeper examination (consolidation and pleural effusion). Although there is no best way to perform image acquisition in LUS [33], most protocols assess the lungs of the patient in the supine position, by dividing each hemithorax into several areas of study. Common approaches to do this are the use of the three points described in the Bedside Lung Ultrasound in Emergency protocol (BLUE-points) or the division of each hemithorax into six areas of study (Figure 1) [34]. However, the optimal number of sites that need to be scanned in LUS is currently debatable, with recommendations ranging from 4 to 28. Interestingly, a recent pilot sub-analysis on the Simple Intensive Care Studies-II (SICS-II) cohort, a prospective study designed to assess the diagnostic and prognostic value of clinical examination and critical care ultrasonography, suggested that increasing the number of scanned areas to more than six is more time consuming and may not provide further diagnostic information [35,36].

**Figure 2:**
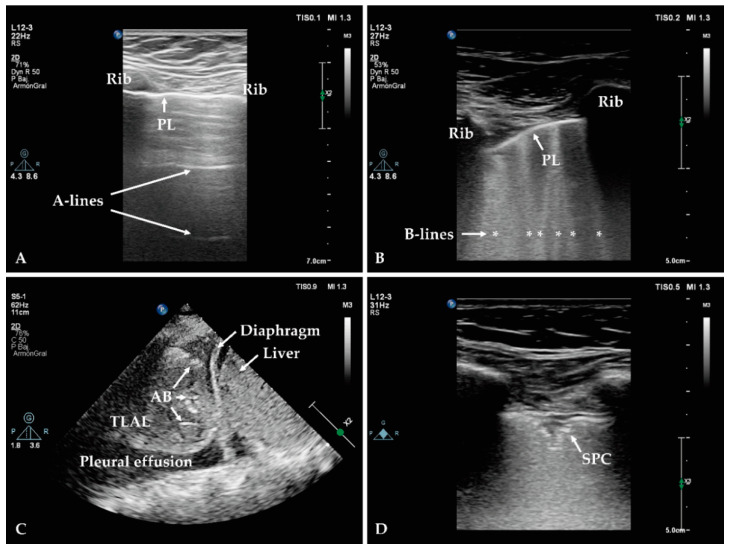
Common signs and artefacts in LUS. (**A**) Normally aerated lung parenchyma. The pleural line (PL) can be recognised as a hyperechoic horizontal line, surrounded by two ribs (bat sign). A-lines are reverberation artefacts which can be visualised as equidistant motionless horizontal lines. Note lung sliding is a dynamic sign and cannot be visualised in a static picture. (**B**) Partially aerated lung parenchyma. Abnormal presence of fluid in the lung parenchyma is responsible for the presence of B-lines (*), which are beam-like hyperechoic vertical artefacts arising from the PL. Note B-lines always reach the edge of the image and erase A-lines. (**C**) Completely de-aerated lung parenchyma. Consolidation originates a tissue-like appearance of the lung (TLAL), inside which air bronchogram (AB) might be visualised as hyperechoic images. (**D**) Subpleural consolidation (SPC). Subpleural consolidations are defined as small (<2 cm) rounded or triangular-shaped hypoechoic areas with ill-defined hyperechoic limits, in contact with the PL.

As defined by Lichtenstein, all signs in LUS come from the pleural line [37]. The pleural line can be visualised by scanning across two ribs with the intervening intercostal space with a high-frequency linear probe. The pleural line originates from the fluid-air interface between the chest wall and the lung, and can be recognised as a hyperechoic horizontal line surrounded by two ribs (“bat sign”) (Figure 2A) [38]. Tidal ventilation is responsible for lung expansion, generating movement of the visceral pleura against the parietal pleura, which can be visualised during LUS examination as a sparkling movement of the pleural line (“lung sliding”) [39]. In the healthy subject, the pleural line generates reverberation artefacts consisting of equidistant motionless horizontal lines (A-lines). A normal lung aeration requires the presence of lung sliding and A-lines (Figure 2A) [40,41]. Abnormal presence of fluid in the lung parenchyma, as it occurs in pneumonia and other conditions, generates an air-fluid interface which is responsible for beam-like hyperechoic vertical artefacts arising from the pleural line (B-lines). B-lines move along with lung sliding, reach the edge of the image and erase A-lines (Figure 2B) [42]. Complete loss of aeration caused by a consolidation leads to a tissue-like appearance of the lung, inside which air bronchogram might be visualised as hyperechoic images (Figure 2C). The features of air bronchogram provide useful information for differential diagnosis: dynamic air bronchogram is consistent with consolidation, which is common in pneumonia, and rule out obstructive atelectasis [43].

LUS is a promising tool for the diagnosis of HAP and VAP. Common findings in LUS in patients with pneumonia include thickened pleural line, irregular pleural line (“shred sign”), diminished lung sliding, subpleural consolidations (Figure 2D), consolidation with tissue-like appearance of the lung, presence of dynamic air bronchogram, and concomitant interstitial pattern (characterised by the presence of several B-lines, generated by the inflammatory component of the interstitium). A recent systematic review and meta-analysis by Staub et al. concluded that small subpleural consolidations and dynamic air bronchograms were the most useful sonographic signs for the diagnosis of VAP in a clinically consistent scenario [44]. Although none of these ultrasonographic signs are exclusive of pneumonia, especially in patients in the ICU, where pre-existence of lung disorders is frequent, identification of new subpleural consolidations or consolidation with dynamic air bronchogram on the day of clinical suspicion of VAP showed high specificity for the diagnosis [44]. Emergence of subpleural consolidations seem to be the earliest sonographic sign of VAP [44]. Of note, the presence of any of these signs without clinical symptoms of VAP, is not sufficient to make the diagnosis, but should prompt the physician to look for symptoms in the following days [44].

The combination of LUS findings and clinical information into predictive models have shown to improve diagnostic performance [15]. The VAP LUS score (VPLUS) combines the presence of purulent secretions (1 point), positivity of tracheal aspirate culture (1 point) and LUS findings (1 point if subpleural consolidation is present and 2 points if dynamic air bronchogram is present), yielding 71% sensitivity and 69% specificity for the diagnosis of VAP when VPLUS ≥ 2 points [45]. The Chest Echography and Procalcitonin Pulmonary Infection Score (CEPPIS) is a modified version of the CPIS in which CXR is replaced by LUS and white blood cell count is replaced by procalcitonin, retaining the rest of the items (temperature, tracheal secretions, oxygenation and endotracheal aspirate quantitative culture) [46]. A retrospective pilot study reported that VAP was better predicted by a CEPPIS > 5 points, outperforming CPIS > 6 points in VAP diagnosis (sensitivity of 80.5% and 39.8%, respectively, and specificity of 85.2% and 83.3%, respectively) [46]. The diagnostic performance of the combination of LUS with procalcitonin was recently assessed by Zhou et al., improving sensitivity to 81.3% and specificity to 85.5%, with a better area under the receiver operating characteristic (ROC) curve for the diagnosis of VAP than any of the diagnostic tests alone [47]. A systematic review and meta-analysis performed by Xia et al. revealed a pooled sensitivity of 95% and a specificity of 91.3% of LUS for the diagnosis of pneumonia when compared to the combination of CT and clinical presentation as the gold standard (to be noted that the study included not only NP, but also community-acquired pneumonia (CAP)) [48].

Some limitations may arise when using LUS for the diagnosis of NP. Firstly, image acquisition and interpretation are operator-dependent, therefore requiring training and experience, even though the learning curve is steep. Secondly, sonographic signs are very sensitive but unspecific, so the clinical correlation is of paramount relevance, as many concurrent disorders may lead to misinterpretation of the findings (acute respiratory distress syndrome, pulmonary oedema, etc.). Thirdly, most sonographic signs are derived from the pleural line, which is a superficial structure; therefore, LUS is of limited value for the identification of lesions located deep in the lung parenchyma. Fourthly, some common ICU conditions may limit image acquisition, including severe obesity, presence of thoracic drains or large bandages, or subcutaneous emphysema. Lastly, no large, prospective studies have addressed the application of LUS for NP diagnosis [15,49].

Despite the limitations mentioned above, important advantages make LUS an attractive diagnostic tool, including the absence of radiation exposure, wide availability, low cost, steep learning curve, the possibility to be performed at the bedside in real-time, and its high level of accuracy [15,49]. LUS may have better sensitivity than CXR, although similar specificity, for the diagnosis of VAP [50]. Nevertheless, it allows clinicians to determine which patients could benefit from further imaging examinations, minimising unnecessary transportation-associated complications [49]. Serial LUS examinations can provide an early diagnosis of HAP or VAP, allow alternative diagnoses to be excluded, and may guide management strategies [45]. Finally, monitoring pneumonia resolution is a potential application of LUS. This can be performed by applying scores to quantify lung aeration at different points in time [32,41]. Improvement of lung aeration observed with LUS is significantly correlated with quantitative reaeration demonstrated by CT scan [45].

#### 2.1.3. Low-Radiation Computed Tomography

**Figure 3:**
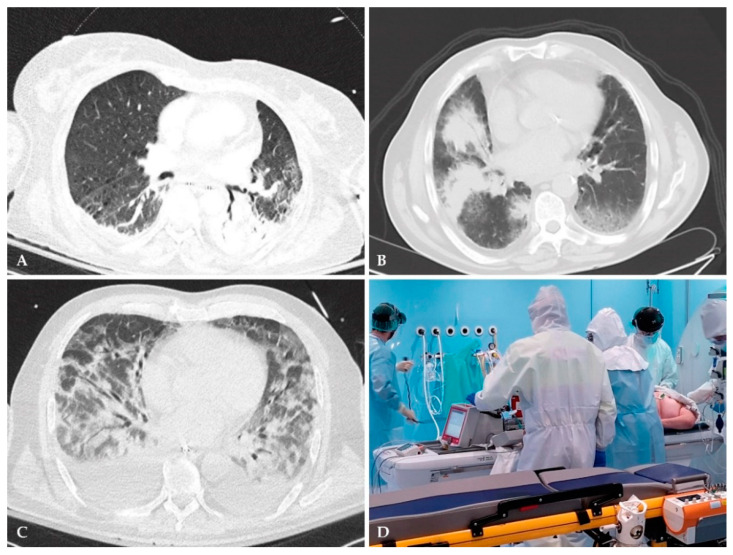
Role of chest CT in the diagnosis of nosocomial pneumonia. (**A**) CT scans can accurately differentiate between atelectasis versus pneumonia compared to CXR, especially among critically ill patients. The left lower lobe retrocardiac consolidation, with air bronchogram, consistent with nosocomial pneumonia, was not visualized on portable CXR, but manifested on CT. (**B**) CT scan may reveal mild infiltrates that are usually missed with conventional CXR. While right lung consolidation shown on this image was visible on CXR, CT allowed for better characterization and revealed a mild infiltrate on the left lower lobe. (**C**) A wide range of lung pathologies may have similar appearances on CT scan. This image illustrates the difficulty in establishing a differential diagnosis in a patient with acute respiratory distress syndrome (ARDS), with suspected VAP. (**D**) In-hospital transfer of critically ill patients represents a logistical challenge with potential risks. This image depicts the transfer of a patient with COVID-19 on extracorporeal membrane oxygenation (ECMO) support to a CT scanner, to rule out VAP.

CT scan remains the gold-standard imaging modality for picking-up lung pathologies and can easily and accurately differentiate between atelectasis versus pneumonia compared to CXR, especially among critically ill patients (Figure 3). Although the sensitivity of chest CT is superior to CXR in picking up pulmonary infections, even mild infiltrations that are usually missed with conventional CXR, its utility is limited by some drawbacks [15]. Firstly, in spite of its exquisite sensitivity, chest CT has low specificity; although a negative imaging practically excludes pulmonary infection, a positive finding does not necessarily advocate pneumonia as many other lung pathologies may have similar appearances on CT scan (Figure 3) [15]. Therefore, the clinical picture alongside conventional and novel diagnostic modalities (as described here) play a pivotal role in establishing the diagnosis of NP. Another disadvantage of CT scans is the logistic challenge of patient transport to the CT scanner. Transferring critically ill patients outside the ICU poses a serious risk to them, even within hospital premises, such as airway loss or displacement, pneumothorax or atelectasis (the risk increases 2–3 times in patients undergoing an in-hospital transport) and hemodynamic complications (Figure 3) [51]. It should be highlighted that portable CT scanners overcome these hazards and make CT scans feasible even for the most unstable critically ill patients, however, they are not widely available across all institutions and their application within ICU departments exposes both staff and patients to unnecessary radiation [15]. Finally, a serious disadvantage of CT scan is the radiation exposure, especially when serial scans are needed to follow up the clinical course of patients [52].

To reduce the radiation exposure, low dose or LRCT scanners have been employed and essentially may change the imaging landscape in pneumonia, as they have a radiation exposure close to the conventional CXR (LRCT = 1–1.5 mSv versus CXR = 0.1 mSv) [15,53]. However, it should be noted that evidence of their diagnostic accuracy within critical care settings is still scarce, and most data is extrapolated from patients with CAP.

A single-centre, prospective study by Prendki et al., compared the LRCT results to CXR in 200 elderly patients (>65 years old) with suspected NP or CAP who were treated with antimicrobials; the patients underwent both imaging modalities within 72 h of inclusion [54]. The authors found that after LRCT, the estimated probability for pneumonia (initially made by treating clinicians before and after the LRCT scan using a Likert scale and subsequently rated by adjudication committee), changed in 90 patients (45%), of which 60 (30%) were downgraded and 30 (15%) were upgraded [54]. Compared with the reference standard as defined by the adjudication committee, 16 patients were correctly reclassified after CT; moreover, the vast majority of those with an intermediate probability (81%) re-scaled after LRCT [53,54]. Contrarily, only 23% of patients rated with high pneumonia likelihood, changed probability after LRCT [53,54]. Therefore, LRCT may be reserved for those with an intermediate probability for pneumonia [53].

Finally, ultralow radiation CT (ultra LRCT) is a novel modality that has a radiation exposure comparable to CXR [55]. Several studies on phantoms and in patients have reported that chest ultra LRCT had sensitivity comparable to LRCT and standard CT in identifying lung pathology [55]. A recent study comparing chest ultra LRCT to CXR (2 CXRs, posteroanterior & lateral) reported almost similar results of the two modalities in terms of speed (<3 min versus <2 min, respectively) and radiation exposure (effective dose 0.071 mSv versus and 0.040 mSv, respectively), while it added value, decreasing the false-positive and false-negative CXR results [55]. However, the cohort did not consist of patients with suspected NP but outpatients with varying lung pathologies; studies including patients with HAP and VAP are needed to assess the utility of ultra LRCT in the diagnostic approach of NP.

Reduced radiation of both LRCT and ultra LRCT scans gives a great advantage in their use and may be valuable assets in the diagnosis of NP; however, this remains to be proven with well-designed large-scale trials.

### 2.2. Aetiological Diagnosis

#### 2.2.1. Conventional Cultures

Conventional aetiologic diagnosis in non-intubated patients relies on sputum samples, while in intubated patients relies on endotracheal aspirates (ETA) and semi-quantitative/quantitative cultures or on invasive quantitative cultures obtained by protected specimen brush or bronchoalveolar lavage (BAL) [1,2]. However, not only may culture methods fail to detect important pathogens due to the administration of empirical antibiotics or stringent growth requirements, but also it may be difficult to distinguish whether the detected organisms are colonisers or actual pathogens [17,56]. Coupled with antimicrobial susceptibility testing, this traditional aetiologic diagnostic approach requires approximately 48 h to 72 h from sample acquisition to result delivery [17]. Until definitive results are available, treatment approach is empirical with administration of broad-spectrum antibiotics to cover the potential pathogens. However, with the emergence of multi-drug resistant organisms (MDROs), especially within intensive care settings, new strategies need to be implemented in order to reduce the pathogen identification time (sampling-to-results time) and achieve faster initiation of appropriate treatment or faster switch to targeted treatment, thus reducing the use of broad-spectrum antibiotics and better compliance with antimicrobial stewardship programs.

A technique that reduces the time of availability of results of the cultures is based on matrix-assisted laser desorption/ionisation time-of-flight mass spectrometry (MALDI-TOF-MS) [57]. MALDI-TOF-MS provides microorganism identification, subtyping and antibiotic susceptibility testing [58]. It is able to identify a large number of targets simultaneously and has been used to reduce the time needed for microbial identification and strain typing in culture-retrieved isolates, becoming an essential tool in many microbiological laboratories over the last decade [57,59]. MALDI-TOF-MS is a protein/peptide-based diagnostic method that relies on the molecular mass of all cellular proteins to determine the characteristic profile of the pathogen [60]. In a study of over 1000 bacterial isolates, MALDI-TOF-MS demonstrated a sensitivity of 95% and a specificity of 84.1% for sample identification [61]. It requires only six minutes to identify each isolate, but of note, it can only use isolates from cultures; despite the initial cost to acquire the equipment, it has been reported to be cost-effective [61,62]. A quasi-experimental study reported that MALDI-TOF-MS improved time to effective antibiotic therapy and optimised antibiotic therapy [63]. A pre-/post-implementation study has shown MALDI-TOF-MS to decrease the length of ICU stay after analysing BAL fluid (*p* = 0.027) [64]. It is clear that MALDI-TOF-MS has significantly accelerated the pathogen identification time and is expected to play an even more important role in the future in microbiology, but it still relies on the traditional, relatively “slow” culture-based techniques [58]. Moreover, it should be noted that, although it allows for quick identification of the species involved (with rare exceptions of poor discrimination or misidentifications between species with inherent similarities), this is not always the case for the antibiotic susceptibility results.

#### 2.2.2. Syndromic Rapid Multi-Pathogen PCR Panels

Contrarily to conventional cultures, novel nucleic acid amplification techniques that are applied directly to raw clinical samples, surpass the stage of pathogen culturing and, therefore, expedite even further, the time required for microbiological diagnosis. The utilisation of molecular techniques, such as syndromic rm-PCR panels, has introduced a new era in the microbiological diagnosis facilitating the early administration of appropriate treatment or the early switch from broad-spectrum empirical to targeted antimicrobial treatment for NP. These techniques have the advantage of identifying multiple targets from a “raw” sample in a timely manner, including microorganisms which are fastidious and pathogens that are not retrieved by conventional cultures, when antimicrobials have already been commenced and perturb their growth. It should be highlighted, however, that the sensitivity of the current rapid test bears the risk of leading to overuse of antibiotics. More studies are needed to further compare their sensitivity/specificity to conventional cultures, as well as validate their clinical benefit.

We will review the novel commercially available syndromic rm-PCR panels, and briefly present other. promising rapid molecular diagnostics test for NP.

(1) BioFire^®^ FilmArray^®^ Pneumonia Panels

BioFire^®^ FilmArray^®^ Pneumonia Panel (BPP)(bioMérieux SA, Marcy-l’Étoile, France) is a Food and Drug Administration (FDA)-cleared syndromic rm-PCR that simultaneously identifies 33 targets: 15 typical and three atypical bacterial pathogens, eight respiratory viruses and seven genetic markers of antimicrobial resistance in BAL/mini-BAL, tracheal aspirates and expectorated sputum specimens [17]. Several of the 18 bacteria included in the panel are from the most commonly implicated pathogens in NP. This assay requires two-minutes hands-on time and about one hour turn-around time, therefore operating as a point-of-care test for rapid detection of NP pathogens [17,65,66]. Similarly to BPP, the BioFire^®^ FilmArray^®^ Pneumonia Panel plus (BPP plus) manufactured by the same company, identifies the same targets along with MERS-CoV virus [67]. Of note, both panels provide semi-quantitative results for the 15 typical bacterial targets (including *Acinetobacter calcoaceticus-baumannii (Acb)* complex, *Enterobacter cloacae* complex, *Klebsiella* spp. and *Pseudomonas aeruginosa*) which helps in the differentiation between colonisers and actual pathogens [66,67].

According to the manufacturing company, BPP has an overall sensitivity of 96.2% and 96.3% and a specificity of 98.3% and 97.2% in BAL and sputum samples, respectively [66]. A prospective observational study among eight clinical sites in the United States by Murphy et al., evaluated the performance of BPP and BPP plus compared to standard of care cultures, quantitative reference cultures and other molecular methods in 836 sputum and 846 BAL specimens, found an overall sensitivity for sputum samples between 75–100% and for BAL 85.7–100%, while specificity for all samples ranged from 88.9—99.5% [65]. Specifically, for *Acb* complex species (common pathogens of NP), the sensitivity and specificity of BPP in sputum samples ranged from 80–90.9% and 97.8–98%, respectively, depending on the comparator method [65]. Similarly, BPP yielded sensitivity and specificity rates as high as 91.3–100% and 94.6–98.9%, respectively, for *Klebsiella pneumoniae* in both types of examined clinical samples [65]. As for the identification of genes encoding antimicrobial resistance, the positive and negative percent agreement (PPA and NPA) rates were 80–100% and 91.4–100%, respectively [65]. In total, the panel detected 15 routinely encountered Gram- and Gram-negative pathogens for pneumonia, with a sensitivity rate of >95% for ten of these analyses in both BAL and sputum, while the other five organisms (*Haemophilus influenzae*, *E. cloacae* complex, *Klebsiella aerogenes*, *Acb* complex and *K. pneumoniae*) had sensitivities ranging from 75–91.7% [65]. Specificity for all targets in both specimen types was >91% and compared to a quantitative reference culture, false negatives were uncommon [65]. However, these results were drawn from clinical specimens taken not only from patients with NP, but from a wide array of patients, including paediatric patients, outpatients and patients visiting the emergency department [65].

A retrospective, single-centre study conducted by Yooet et al. on 100 sputum and ETA specimens from hospitalised patients compared the results of routine cultures and antimicrobial susceptibility testing with BPP results in both typical respiratory bacterial pathogens detection and antibiotic resistance profiles [17]. Of 99 specimens with interpretable results, BPP yielded positive results in 73 (73.7%), compared with culture which yielded 65 (65.7%) positive results [17]. BPP detected one pathogen in 29 specimens, two pathogens in 23 specimens, three pathogens in 12 specimens and ≥ four pathogens in nine specimens [17]. In contrast, culture-based techniques detected one pathogen in 59 specimens and two pathogens in the remaining six specimens [17]. Of 69 specimens that exhibited significant amounts in culture, 67 (97.1%) was found to have ≥106 copies/mL of bacterial nucleic acids by BPP, and among 41 specimens that did not exhibit significant bacterial growth in culture, BPP detected 26 that gave negative results and 15 demonstrated positive results with ≥104 copies/mL [17]. Additionally, of 18 specimens with species found resistant by routine antimicrobial susceptibility testing, BPP detected antimicrobial resistance markers in 17 (94.4%) of them [17]. Overall, BPP demonstrated sensitivity and specificity of 98.5% and 76.5%, respectively and is estimated to have guided antibiotic treatment in 50% (23/46) of suspected pneumonia cases [17].

Similarly, a single-centre, prospective study by Edin et al. evaluating the BPP plus compared to standard diagnostic culture-based techniques among 84 clinical samples (sputum, ETA and BAL) taken from both ICU and non-ICU patients with suspected lower respiratory tract infection, found that BPP plus detected a pathogen (including *H. influenzae*, *S. aureus*, *Serratia marcescens*, *Mycoplasma pneumoniae*, and *P. aeruginosa*) that was not covered by the administered empirical antibiotics in 15 patients (25%) [68]. Moreover, the BPP plus demonstrated increased detection of viruses and atypical bacterial pathogens [68]. The overall PPA and NPA for BPP plus were 86% and 67.7%, respectively [68]. Although this observational study limits the evaluation of the panel’s efficacy in diagnostics and management, it was estimated that clinical decisions relating to isolation measures could potentially be influenced in 30% of patients [68].

It should be emphasised that the patient population within ICUs differs significantly from other hospital departments and, certainly, outpatient settings, as many patients are receiving antibiotics (or had a course of antibiotics recently administered), are frequently colonised with MDROs and pneumonia symptoms and signs are often subtle, making the interpretation of any diagnostic test more challenging. However, data regarding the performance of BPP and BPP plus solely in ICU patients is limited. A small single-centre prospective study by Lee et al. evaluated the performance of BPP on 59 ETA and BAL specimens taken from 51 adult patients admitted in medical ICU with respiratory failure [69]. This study demonstrated an overall PPA and NPA rate of 90% and 97.4%, respectively, for detecting bacterial pathogens compared to conventional culture-techniques [69]. Not surprisingly, BPP resulted in more than one analyte detection per single specimen in 42.3% of samples, and multiple detections were higher in sputum (92.9%) than in BAL and bronchial wash (7.1%) specimens. Additionally, viruses were detected in 27.1% of specimens, which would have been neglected with conventional culture-based techniques [69]. The authors reported that BPP results could have led to alteration of the prescribed antibiotics in 40.7% of patients [69]. Moreover, Yugueros-Marcos et al. conducted a prospective, non-randomised, non-interventional, multi-centre clinical trial, whereby a total of 117 BAL specimens from suspected VAP patients were tested with the BPP and the semi-quantitative results produced by the panel (using an agreed positivity threshold) were compared to conventional cultures [70]. Overall, PPA and NPA for bacterial isolates were 89% and 95.9%, respectively [70]. A total of 74 (39%) identification results were discrepant between both techniques; although in 26 (14%) cases the same microorganism was detected, the results were considered as discrepant due to the different positivity thresholds between techniques [70]. It should be noted that the 11 falsely positive samples for BPP, could be attributed to negative cultures due to the administration of antibiotics therefore, leading to an overall concordance of 93.1% PPA and 98.2% NPA [70].

(2) Curetis Unyvero multiplex PCR Panels

Syndromic rm-PCR panels for HAP/VAP have also been developed by Curetis (Curetis GmbH, Holzgerlingen, Germany; Curetis, an OpenGen Group, Gaithersburg, MA, USA): the Unyvero P55 which has been discontinued by the company, the Unyvero Lower Respiratory Tract (LRT/LRT BAL) and Unyvero Hospitalised Pneumonia (HPN) [71]. The Unyvero P55 Pneumonia panel, capable of identifying 20 causative agents of lower respiratory tract infections (LRTI) and 19 antibiotic resistance determinants, was compared to routine microbiological culture and antimicrobial resistance (AMR) diagnostics, in a double-centre study by Ozongwu et al. [72]. The study was conducted in fresh respiratory samples (<48 h old) taken from inpatients with HAP (n = 44), VAP (n = 14) and CAP (n = 27), and the samples consisted mainly of sputa and ETAs [72]. Unyvero P55 agreed with culture results in 57 (67%) of 85 analysed specimens, of which the same organisms were identified in 12 specimens [72]. Negative results were concordant in 27 (31.7%) specimens, while Unyvero P55 identified at least one additional organism in 18 (21.2%) specimens [72]. The overall sensitivity and specificity rates were 88.8% and 94.9%, respectively, with lowest sensitivity being for *Streptococcus pneumoniae* (33.3%) [72]. In terms of AMR, Unyvero P55 detected 18 occurrences of relevant resistance markers, whilst routine microbiology identified resistance in ten isolates [72]. However, compared to BPP, Unyvero P55 assay was found to have a lower sensitivity (63.8–88.8% vs. 98.5%) and take longer for the sample-to-result time (5 h vs. 1 h) [17,72,73].

Three studies have tested the Unyvero P55 and/or HPN panels in samples taken from patients within critical care settings. Both panels detect the same targets with the exception of *Enterobacter aerogenes* which is only detectable by Unyvero P55. Gadsby et al. conducted a single-centre trial comparing the Unyvero P55 panel with an in-house PCR and routine cultures in 74 BAL specimens collected from ICU patients, among which 29 (39.2%) had suspected VAP [73]. The authors demonstrated sensitivity and specificity to be as low as 56.9%/58.5% and 63.2%/54.8% for Unyvero P55 and in-house PCR panels, respectively, whilst sensitivity for in-panel targets was 63.5% and 83.7%, respectively [73]. Not surprisingly, additional pathogens were detected in both PCR assays compared to routine cultures [73]. In relation to AMR gene identification, Unyvero P55 showed very low sensitivity (18.8%), whereas specificity rate was very high (94.9%) [73]. Another study by Peiffer-Smadja et al., conducted in 95 BAL or plugged telescoping catheter samples from ventilated ICU patients with VAP or HAP, compared the performance of Unyvero HPN panel to culture-based and AMR diagnostics [74]. Whilst the overall sensitivity and specificity was 80% and 99%, respectively, Unyvero HPN had a higher sensitivity for Gram negative bacteria (90%) than for Gram positive cocci (62%) (*p* = 0.005) [74]. Importantly, the authors estimated that Unyvero HPN results could have led to an earlier initiation of an effective antibiotic in 20 (21%) patients, whilst in 37 (39%) patients, it could have guided an earlier de-escalation, including ten carbapenem-based therapies that could have been deescalated within hours [74]. Finally, Unyvero HPN detected two cases of severe *Legionella* that were also confirmed with cultures [74]. The third study conducted by Luyt et al. prospectively utilised Unyvero P55 or Unyvero HPN cartridges in 93 BAL samples from patients with suspected or confirmed VAP and compared the results to those of conventional microbiological techniques [71]. The authors reported that the rm-PCR assays used correctly identified pathogens in 68 (73%) proven VAP episodes, whilst their results were discordant in 25 (27%) episodes (no pathogen was detected in 11 samples and in six samples the Unyvero “overdetected” a pathogen that was not detected by culture-based techniques) [71]. The remaining results concerned pathogens responsible for VAP that were not included in the multiplex panel or grew at a non-significant level in culture [71]. Regarding resistance genes, the Unyvero failed to detect them in 71% of VAP episodes [71]. Therefore, the overall sensitivity and specificity rates for pathogen detection were 77.4% and 14.3%, respectively, whereas the respective rates for resistance detection were 46.3% and 82.7% [71].

VAPERO (NCT03711331) is an ongoing RCT that intends to include 160 participants to measure the impact of Unyvero multiplex PCR tests on the adjustment of antimicrobial therapy in patients with suspected VAP/HAP that require mechanical ventilation, compared to standard care [75].

Finally, a very recent, multi-centre,. prospective study by Enneet et al. (INHALE WP1 Study Group) conducted in 15 United Kingdom (UK) hospitals, assessed the performance of the two previously described rm-PCR panels (BPP and Unyvero Pneumonia Panel) compared to conventional cultures in ICU patients with NP (VAP or HAP) who were about to start antibiotics or change antibiotic treatment [76]. The researchers reported that among 652 eligible samples, PCR panels identified pathogens in considerably more samples compared with routine culture-based techniques (60.4% and 74.2% for Unyvero and BPP, respectively, versus 44.2% for conventional microbiology cultures) [76]. BPP had a sensitivity of 91.7–100.0% and a specificity of 87.5–99.5%, for common HAP/VAP pathogens, while Unyvero had sensitivity of 83.3–100.0% except for *K. aerogenes* (50.0%) and *S. marcescens* (77.8%), and a comparable with BPP specificity of 89.4–99% [76]. Moreover, both panels detected more organisms per sample than routine culture and frequently in agreement with each other [76]. Finally, PCR panels detected more high-consequence antimicrobial resistance genes than would have been identified by routine antimicrobial susceptibility testing [76].

In Table 1, a summary of the characteristics of commercially available, syndromic multiplex PCR panels used for the aetiological diagnosis of NP, i.e., suitable types of respiratory samples, pathogens and AMR genes detected, and time to results.

(3) Other syndromic rapid multi-pathogen PCR panels

A novel 16S (rRNA) pan-bacterial PCR assay tested BAL samples taken from patients from the VAPRAPID randomised, controlled, multi-centre trial (NCT01972425), to determine its utility for a rapid (within 4–5 h) microbiologic confirmation of VAP [77]. Results of the 16S PCR test using cycles to cross threshold (Ct) values, demonstrated that the area under the ROC curve was 0.94 (95% CI 0.86 to 1.0, *p* < 0.0001) in a derivation cohort, 15% of whom had confirmed VAP, and 0.89 (95% CI0.83 to 0.95, *p* < 0.0001) in a confirmation cohort, 28% of whom had confirmed VAP [77]. The authors concluded that 16S pan-bacterial PCR can be used to quickly exclude VAP in suspected cases, but further studies are needed for the assessment of its utility [77].

Recently, in a UK centre, a novel rapid multiplex syndromic panel (custom designed multi-pathogen TaqMan Array Cards, TAC; Thermo Fisher Scientific) that detects 52 different respiratory pathogens (39 bacteria, 4 fungal strains and 9 viruses) in BAL samples, was developed and implemented in mechanically ventilated patients with suspected pneumonia [78]. The selection of microorganisms for TAC was based on the microbial flora of the ICU along with the literature concerning pathogens of severe pneumonia [78]. The primary outcome of the study was the concordance of TAC with the reference standard of conventional cultures in combination with metagenomic sequencing, while the primary co-outcome was time to result compared to validated conventional cultures [78]. Overall, the rapid test demonstrated high sensitivity and specificity rates (92% and 99%, respectively) compared to conventional lower respiratory cultures and metagenomic sequencing [78]. The results of this rapid test were available in a median of 61 h (IQR 42–90) earlier than the culture-based techniques and, importantly, they led to alteration of clinical management in 53% of the patients (de-escalation, in 64%, spectrum increase in 27%, and alternative diagnosis assessment in 9%) [78]. Additionally, the group that the TAC diagnostic approach used had significantly more antibiotic-free days than the comparator [78]. The broad range of detected pathogens, along with its customisability, makes multi-pathogen TAC a very promising tool. Based on the above results, i.e., high sensitivity, faster results, and measurable impact on patient management, this syndromic diagnostic approach to severe pneumonia with TAC was adopted as a routine practice in the clinical service of the institution that conducted the study (liver/general ICU, Addenbrooke’s Hospital, Cambridge, UK) [79]. Moreover, very recently, the same centre reported the results of a retrospective study that used a combination of conventional lower respiratory cultures and multi-pathogen TAC to compare the incidence of microbiologically confirmed VAP in mechanically ventilated, critically ill patients with or without COVID-19 [79]. In addition, a 16S RNA analysis was used in a subset of samples to determine the lung microbiome [79]. Using a previously defined positivity threshold of ≤32 cycles (that corresponds to a growth of ≥10^4^/CFU/mL in conventional culture-based methods), the authors reported that COVID-19 patients were significantly more likely to develop VAP (Cox proportional hazard ratio 2.01, *p*  =  0.0015 [78]. Interestingly, the increased risk for VAP development in COVID-19 patients could not be explained by the patterns of pulmonary dysbiosis, as both COVID-19 and non-COVID-19 groups had similar pulmonary microbiome composition [79]. It is noteworthy that the concordance between conventional cultures and the rapid molecular testing results were high; moreover, an additional number of microorganisms were identified by the molecular test [79]. TAC is not yet commercially available.

Several other multiplex PCR assays have been developed for the detection of respiratory pathogens [57]. However, their usefulness in critical care settings has not been established yet. The commercially available respiratory panel by Fast track Diagnostics (Esch-sur-Alzette, Luxembourg, Luxembourg), the FTD Respiratory pathogens 21, detects mainly viruses as well as *M. pneumoniae*, therefore important causative bacteria of NP are not included [80]. Similarly, the RespiFinder^®^ SMART 22 (PathoFinder^®^, Maastricht, Limburg, The Netherlands) and VERIGENE^®^ Respiratory Pathogens Flex Test (Luminex^®^, Austin, Texas, USA) detect only viral and atypical pathogens [81,82,83].

#### 2.2.3. Other Rapid Molecular Diagnostics

The GeneXpert^®^ (Cepheid^®^, Sunnyvale, CA, USA) is a family of systems that allows for automated molecular diagnostics. It helps identify mechanisms of resistance and is capable of delivering most test results in one hour, including sample preparation time, faster than alternative technologies such as enzyme immunoassays [84]. Using advanced microfluidics, the process of nucleic acid extraction, amplification and detection is performed within each single-use cartridge, minimising the risk of cross contamination [84,85]. The GeneXpert CarbaR is capable of detecting carbapenem resistance genes (*K. pneumoniae* carbapenemase (KPC), oxacillinase-type carbapenemase (OXA-48, OXA-181, OXA-232), and metallo-beta-lactamases (MBLs) which include imipenemase MBL-1, New Delhi MBL and Verona integron-encoded MBL) within 48 min [86]. The diagnostic performance of the GeneXpert CarbaR was evaluated using 408 rectal swabs and found to have 100% sensitivity, 96.7% specificity, a positive predictive value of 53.6% and a negative predictive value of 100% [87]. A single-centre, prospective study that compared the performance of the GeneXpert CarbaR panel with standard culture-based antimicrobial susceptibility techniques in a cohort of 20 critically ill patients with abdominal sepsis; two rectal/stomia swabs and two swabs from abdominal drainage fluid were collected by each patient, and each set of rectum/stomia and abdominal drainage fluid swabs was tested with either GeneXpert CarbaR panel or conventional methods (meropenem antimicrobial susceptibility disks followed by E-test) [88]. The overall sensitivity and specificity of the GeneXpert CarbaR panel to detect carbapenem resistance (including genes that are not detected by the panel) were 50% and 93.1%, respectively [88]. However, when comparing the performance of the test only for the five AMR genes detected by the GeneXpert CarbaR, sensitivity and specificity rates were significantly higher (100% and 94.2%, respectively) [88]. Hence, its use is limited by the narrow panel of detected genes and should be guided by the local epidemiology of antimicrobial resistance profiles; the performance and clinical utility of the GeneXpert CarbaR could be augmented by the inclusion of more genes (e.g., OXA-23) and alleles of certain gene families (e.g., OXA-181) [88]. The GeneXpert MRSA/SA, another on-demand molecular test allows for the detection of MRSA or *S. aureus* from a Gram-positive blood culture sample within one hour, and has been shown to reduce the mean length of stay by 6.2 days, allowing for the use of optimal antimicrobial therapy 1.7 days sooner and reducing hospital costs per bacteraemic patient by EUR 15,683 [89]. When compared to semi-quantitative cultures in 79 endotracheal aspirate samples for the detection of *S. aureus*, GeneXpert MRSA/SA assays were found to be 100% sensitive and 100% specific, making it the best tool for the direct detection of *S. aureus* in ETA samples in this comparator study [90]. However, limitations remain as samples are taken from swabs, therefore, like culture-based methods, it is unable to distinguish between colonisers and pathogens.

The advantages and disadvantages of multiplex PCR panels are presented in Table 2.

#### 2.2.4. Volatile Organic Compounds—Electronic Nose

Another promising diagnostic technique for the rapid and non-invasive diagnosis of NP in the future is the analysis of exhaled breath from exogenous and endogenous sources [91]. Exhaled breath contains thousands of volatile organic compounds (VOCs) which reflect biological processes both locally and systemically in the patient [92]. Oxidative stress and inflammation, as well as invading microorganisms produce specific compounds, which can induce alterations in the compositions of VOCs, leading to distinct VOC profiles in exhaled breath [91]. Due to the invasion of harmful microorganisms in the lung, coupled with the body’s defence mechanisms, VOCs have been shown to be present in varying concentrations and compositions compared to patients without VAP [91].

A study conducted in 100 critically ill patients with a clinical suspicion of VAP was conducted to assess the usefulness of VOC analysis as a non-invasive monitoring tool [91]. Exhaled breath samples were collected from ventilated patients directly before BAL was performed, which were measured by gas chromatography-time-of-flight-mass spectrometry (GC-tof-MS) [91]. This resulted in a set of 12 chemically diverse VOCs which have the potential to determine the presence of VAP with an accuracy of 74.2%, sensitivity of 75.8% and specificity of 73.0% [91]. Potential confounders, including haematological underlying diseases and active malignancies were not found to be significantly associated with the VOC profile [91]. Although GC-tof-MS, the current gold standard, is a highly sensitive method to accurately measure trace gases in exhaled air, it is time-consuming and carries a risk of contamination, limiting its use as a point-of-care testing technology for VOC [92,93].

A recent development in these field is the electronic nose technology (eNose), developed by The eNose Company (Zutphen, CA, The Netherlands) an artificial sensor system consisting of a range of chemical sensors that resemble biological olfactory receptors to detect VOCs [94,95]. VOCs attach to the sensor polymer surface and induce swelling of the polymer film, increasing the electrical resistance and generating an electrical signal [95]. These signals can be classified into VOC signatures using algorithms and a database of previously recorded VOC patterns [94,96]. Distinct VOC patterns have the potential to serve as markers of inflammatory, microbial, oxidative and neoplastic conditions [96,97,98]. The eNose technology has been implemented in the food and beverage industry to monitor air quality and to detect explosive and chemical agents in the environment [96,99]. In COPD subjects, the eNose was able to distinguish between those with a viral or bacterial infection and those without an infection [100]. Similarly, eNose technology was used in six case and five control patients with probable or proven aspergillosis, which reported a cross-validated accuracy of 90.9% (*p* = 0.022, sensitivity 100%, specificity 83.3%) [101].

In vitro studies using gas chromatography/mass spectrometry (GC-MS) analysis, have shown that as many as 34 volatile metabolites, including alcohols, aldehyde, esters, hydrocarbons, ketones and sulphur-containing compounds, were released from *S. pneumoniae*, and 28 released from *H. influenzae* [102]. Other in vitro studies have also reported the detection of *S. aureus*, *Escherichia coli*, *P. aeruginosa*, *Moraxella catarrhalis*, and *Mycobacterium tuberculosis* in bacterial cultures [95,103,104,105]. These findings are also mirrored in animal studies which report that VOCs released from the breath of mice with lung infections of *P. aeruginosa* and *S. aureus*, were detectable in cultures in vitro [106,107].

A recent case–control study aimed to develop a standardised protocol for machine learning technique for use in analysing VOCs [95]. Exhaled breath of 61 participants with suspected VAP was collected in the lower respiratory tract to prevent contamination from the air, oral cavity and dead space air and to increase the concentration of VOCs collected [95]. The mean number of pathogens detected in respiratory cultures of participants in the case group was 1.52, with *K. pneumonia* being the primary pathogen isolated in (42.4%), followed by *S. aureus* (15.15%) and *Stenotrophomonas maltophilia* (15.15%) [95]. The study demonstrated high diagnostic accuracy in predicting VAP (mean accuracy 0.81 ± 0.04, sensitivity was 0.79 ± 0.08, specificity 0.83 ± 0.00, positive predictive value 0.85 ± 0.02, negative predictive value 0.77 ± 0.06) [95].

Contrarily, a similar study conducted in 72 patients demonstrated a lack of sensitivity and specificity of eNose in the diagnosis of VAP [108]. When patients with a BAL confirmed diagnosis of VAP were compared to those without a clinical suspicion of VAP, the sensitivity was 88% with specificity 66% [108]. When patients with a BAL confirmed diagnosis of VAP were compared to those in which BAL analysis was negative for VAP, the sensitivity was 76% with specificity 56% [108].

Although the emerging technology of eNose has potential to be a non-invasive, cheap, fast and efficient technique to diagnose HAP/VAP, it is important to note that underlying diseases and co-infections may decrease the discrimination ability and will require further research to investigate these effects. Currently, a non-randomised clinical trial (NCT02652247) is aiming to recruit 350 participants to investigate the effectiveness of exhaled breath condensate fluid for early, non-invasive detection of VAP in critically ill or injured patients [109].

## 3. Novel Approved Antibiotics for Nosocomial Pneumonia

### 3.1. Ceftobiprole Medocaril

Ceftobiprole medocaril, trade name Zevtera™/Mabelio™, marketed by BasileaPharmaceutica (Basel, Switzerland), is a 5th-generation cephalosporin approved by the European Medicines Agency (EMA) in 2013 for the treatment of bacterial HAP (but not VAP) and bacterial CAP [110].

Similar to other cephalosporins, ceftobiprole exerts its action by binding to penicillin-binding proteins (PBPs), which interferes with cell wall synthesis, inhibiting cell growth and leading to bacterial cell death [111]. It demonstrates potent binding to PBP2a in MRSA, PBP2x in penicillin-resistant *S. pneumoniae*, PBP2 and PBP3 in *E. coli*, and PBP2 in *P. aeruginosa* [111].

Ceftobiprole exhibits an extended spectrum of activity against both Gram-positive and Gram-negative bacteria [111,112]. Concerning Gram-positive microorganisms, this antibiotic is active against streptococci (viridans, β-hemolytic and *S. pneumoniae*, including penicillin- and ceftriaxone-resistant strains), with a minimum inhibitory concentration (MIC_90_) ≤ 0.5 mg/L; coagulase-negative staphylococci (CoNS) and *S. aureus*, with a MIC_90_ ≤ 0.5 mg/L for methicillin-susceptible CoNS and MSSA, 2 mg/L for MRSA and vancomycin intermediate *S. aureus*, and 4 mg/L for methicillin-resistant CoNS. It is also modestly active against *Enterococcus faecalis* (MIC_90_ 4 mg/L), but inactive against *Enterococcus faecium* [113]. Concerning Gram-negative bacteria, ceftobiprole shows a similar spectrum to that of 3rd and 4th generation cephalosporins, with little to no activity against AmpC- and extended spectrum beta lactamase-(ESBL) producing microorganisms, but good activity against *Neisseria* spp., *H. influenzae* and *M. catarrhalis*, with a MIC_90_ < 0.25 mg/L; *E. coli*, *Klebsiella* spp., *Proteus* spp. and *Morganella* spp., with a MIC_90_ ≤ 0.5 mg/L; and *Enterobacter* spp., *Citrobacter* spp. and *Serratia* spp., with a MIC_90_ 1 mg/L [112,114]. Ceftobiprole has a binding profile comparable to that of cefepime and ceftazidime to PBPs in *P. aeruginosa*, with enhanced binding to PBP2 (MIC_90_ = 4 mg/L) [112,113,115]. Atypical bacteria, *Nocardia* spp., *Stenotrophomonas* spp., *Acinetobacter* spp., and *Burkholderia* spp. are resistant to ceftobiprole [110,112,115,116].

Ceftobiprole has been shown to have a low potential to select for resistance [111]. Even though staphylococci have a great ability to develop resistance to a wide range of antimicrobial agents in the clinical setting, in vitro studies suggest that MRSA has a low potential to become resistant to ceftobiprole [117]. The most common changes leading to in vitro resistance of MRSA to ceftobiprole are mutations in the mecA gene resulting in changes in the transpeptidase domain of PBP2a and in the non-penicillin-binding domain, and mutations in different PBPs, particularly those leading to overexpression of PBP4 [118]. Other mutations have been involved in the development of 5th-generation cephalosporins resistance, including ClpX endopeptidase, PP2C protein phosphatase, transcription terminator Rho, and GdpP phosphodiesterase [119]. A recent surveillance study in the United States found 0.3% of MRSA non-susceptible to ceftobiprole in blood isolates [120]. Another study in Italy found 12% of ceftobiprole resistance among the MRSA population (only mecA producers) [121]. In the light of these results, it is important to assess susceptibility to ceftobiprole in order to avoid therapeutic failure and selection of resistant strains.

Ceftobiprole exhibits a linear, time-independent pharmacokinetic profile [122]. Pharmacokinetic parameters after administration of a 500 mg dose of ceftobiprole by intravenous (IV) 2-h infusion: peak plasma concentration (C_max_) 29.2 mg/L; area under the concentration-time curve (AUC) 90 mg × h/L; plasma protein binding 16%; volume of distribution (Vd) 18 L; minimal hepatic metabolism (4%), with no interactions with P450 isoenzymes or P-glycoprotein 1; plasma elimination half-life (t_1/2_) 3 h; excretion mostly via the kidneys (80% as active compound) [110,112,116,123]. As with other cephalosporin antimicrobials, the fraction of time during the dosing interval in which the free drug concentration remains above the MIC for the infecting microorganism (*%fT*> MIC), has been shown to be the parameter that best correlates with the efficacy of ceftobiprole [122]. Intrapulmonary penetration of ceftobiprole in healthy subjects was assessed in a phase I study, demonstrating epithelial lining fluid (ELF)/plasma ratio of 25.5% [124], a value consistent with ELF/plasma ratios of other cephalosporins [125]. In an animal model of disease (murine model of pneumonia), penetration of ceftobiprole into the lung tissue was significantly higher (ELF/plasma ratio 68.8%) [124].

The approved dosing regimen of ceftobiprole for adults with bacterial CAP or HAP is 500 mg every 8 h by IV 2-h infusion, for 7–14 days [116]. While no change is needed in patients with deranged liver function, dosage adjustment is required in patients with impaired renal function: 500 mg/12 h for patients with eGFR 30–50 mL/min; 250 mg/12 h for patients with eGFR 15–30 mL/min; 250 mg/24 h for patients with eGFR < 15 mL/min; 250–500 mg/24 h for patients on chronic haemodialysis [116]. Although dosing in patients under continuous renal replacement therapy (CRRT) is not well stablished, a dose of 250 mg/12 h seems to be safe and provide optimal pharmacodynamic target attainment (1- to 4-fold 100% time above the MIC) against MRSA (2–8 mg/L), and also against other common pathogens in pneumonia, according to a case report [126]. The influence of ECMO on the pharmacokinetics of ceftobiprole has not been addressed yet.

The approval of ceftobiprole for the indication of bacterial CAP in hospitalised patients was based on a study which demonstrated non-inferiority of the antimicrobial agent when compared to ceftriaxone plus linezolid (NCT00326287) [127]. The approval of ceftobiprole for the indication of bacterial HAP, excluding VAP, was based on a randomised, controlled, double-blind, phase III, non-inferiority trial in 781 patients with bacterial HAP, including 210 with VAP (NCT00210964, NCT00229008) [128]. This study compared ceftobiprole to ceftazidime plus linezolid for 7–14 days of treatment [128]. Efficacy was assessed as clinical cure at the test-of-cure visit (59.6% for ceftobiprole vs. 58.8% for ceftazidime plus linezolid) [128]. Of note, patients with a baseline culture positive for MRSA showed early improvement (<4 days) in a higher proportion in the ceftobiprole group (94.7% vs. 52.6% in the ceftazidime plus linezolid group) [128]. Clinical cure rates of patients with HAP requiring mechanical ventilation during treatment or developing pneumonia within 48 h after the start of ventilation (thus, not meeting VAP criteria) were also higher in the ceftobiprole group (55.3% vs. 40.5% in the ceftazidime plus linezolid group) [128]. However, non-inferiority was not demonstrated in VAP patients (clinical cure 23.1% for ceftobiprole vs. 36.8% for ceftazidime plus linezolid) [128].

Ceftobiprole has a synergistic effect with daptomycin against *S. aureus* and *E. faecalis* [129]. It inhibits OATP1B1 and OATP1B3 in hepatocytes, with potential to increase plasma concentration of drugs cleared by these pathways (i.e., statins, glibenclamide, bosentan) [110,112,116]. Ceftobiprole may precipitate when mixed with calcium-containing solutions (except with Ringer lactate) [110,112,116].

Most frequent side effects of ceftobiprole are mild and include gastrointestinal symptoms (nausea [2.1%], vomiting [1.6%], diarrhoea [3.1%], dysgeusia [1.3%]), hyponatremia (4.4%) injection site reactions (2.1%) and hypersensitivity reactions (0.8%) [128]. Uncommon side effects (<1%), but potentially more severe, include thrombocytopenia, agranulocytosis, anaphylaxis, *Clostridium difficile* infection, colitis, seizures, agitation and acute kidney failure [116,128].

Ceftobiprole is a 5th-generation cephalosporin with a wide spectrum of antimicrobial activity, representing a good alternative for the treatment of bacterial CAP and HAP, excluding VAP, even in scenarios where MRSA is proven/suspected.

### 3.2. Telavancin

Telavancin (Vibativ™), developed by Theravance Biopharma Ireland Ltd. (Ballsbridge, Dublin, Ireland), is a semi-synthetic, lipoglycopeptide derivative of vancomycin that was approved by the FDA in 2013 for the treatment of bacterial HAP/VAP caused by susceptible isolates of *S. aureus* and complicated skin and skin structure infections (cSSSI) when alternative treatments are not suitable [130].

The label was expanded in 2016 to include patients with concurrent *S. aureus* bacteraemia in cSSSI patients and bacterial HAP/VAP patients after phase III ATLAS and ATTAIN trials, respectively, were conducted [131]. The EMA approved the use of telavancin in 2014 for the treatment of bacterial HAP/VAP caused by known or suspected MRSA NP, when alternative treatments are not suitable, however in 2018, Theravance Biopharma Ireland Ltd. decided to permanently discontinue the marketing of telavancin due to commercial reasons [130,132]. Cumberland Pharmaceuticals Inc. announced its decision to acquire Vibativ from Theravance Biopharma in 2018 [133]. In 2020, Cumberland Pharmaceuticals announced its initiative to expand the availability of Vibativ to treated HAP and VAP resulting from coronavirus infections to help address potential antibiotic shortages [134].

Telavancin was developed from vancomycin by adding a lipophilic side chain and an aminomethylphosphonite group [135]. This structural modification enables enhanced lipophilicity and membrane penetration, increased antimicrobial activity against Gram-positive pathogens and reduced potential for resistance [130]. Similar to vancomycin, telavancin has a glycopeptide core which binds with high affinity to the acyl-D-alanyl-D-alanine terminus of the cell wall precursors, therefore inhibiting peptidoglycan synthesis [136]. In addition, telavancin also binds to bacterial cell membranes, causing membrane depolarisation and disrupting the membrane permeability [136]. This dual mechanism of action is believed to be responsible for the rapid, dose-dependent bactericidal activity, unlike that seen in vancomycin [136]. Owing to its dual mechanism of action, telavancin exhibited a 50% inhibitory concentration of 0.14 µM, approximately 14-fold more potent than vancomycin [137].

Similar to vancomycin, telavancin is active against Gram-positive aerobic and anaerobic bacteria, however characterised by a MIC that is generally two to eight times lower for most organisms [138]. An in vitro study demonstrated telavancin to be active against all Gram-positive species tested with MIC ≤ 1 mg/L in 89% of isolates [139]. The majority of staphylococci isolates, including methicillin-, erythromycin- and moxifloxacin-resistant isolates, were inhibited by 0.125–1 mg/L of telavancin, with two isolates of methicillin-resistant *Staphylococcus epidermidis* being the exceptions with telavancin MICs of 2 mg/L [139]. Specifically, telavancin was active against MSSA and MRSA with a MIC of 0.12–2 and ≤0.06–2 mg/L, respectively [138]. Vancomycin-susceptible isolates of enterococci were inhibited by telavancin at 0.06–1 mg/L, however vancomycin-resistant strains were less susceptible, with telavancin at MICs 0.125–8 mg/L. The majority of the enterococci were erythromycin-resistant with 70% of isolates with erythromycin MICs >64 mg/L and 62% with gentamicin resistance, however all enterococci were susceptible to linezolid with MICs 1–4 mg/L [139]. Against β-haemolytic streptococci, including erythromycin-resistant isolates, telavancin demonstrated high activity with MICs 0.03–0.123 mg/L [139]. Telavancin also demonstrated activity against all isolates of *Listeria monocytogenes* (MIC 0.06–0.125 mg/L) and comparable activity against *Lactobacillus* spp., which are intrinsically resistant to vancomycin, to enterococci with high-level vancomycin resistance [139].

In vitro studies suggest that telavancin has a low potential to select for resistance. In multi-step resistance selection studies, a single stable mutant appeared after 43 days in one MRSA strain out of ten tested, with MIC increasing from 0.25 µg/mL to 2 µg/mL, which did not increase further by 50 days [140]. Significant reductions in susceptibility were not seen in enterococci strains. Single-step mutation frequencies were also lower for telavancin than the spontaneous mutation frequencies of comparators [140]. Resistance to glycopeptides, vancomycin and teicoplanin, results from the substitution of D-Ala-D-Lac for D-Ala-D-Ala in susceptible bacteria, leading to a 1000-fold lower affinity for vancomycin [141]. This is frequently seen in VRE mediated by two related gene clusters vanA and vanB [141]. In contrast to vancomycin, the D-Ala-D-Lac- containing precursor was not detected in the two telavancin-treated vanB-type cultures [141].

The pharmacokinetic profile of telavancin closely resembles vancomycin, with a C_max_ of 76.7 vs. 74.6 mg/L [130]. The half-life of telavancin is moderately higher (6.5 h vs. 4.95 h) with a lower total clearance (1.19 vs. 5.79 L/h), mainly renally excreted with 76% of the dose recovered in urine and 1% in faeces [130]. Generally, telavancin is dosed at 10 mg/kg by IV infusion over 60 min every 24 h for 7–21 days for the treatment of bacterial HAP/VAP [142]. However, due to its renal involvement, dose adjustment is required in patients with renal impairment; once daily intravenous dose of 10 mg/kg with creatinine clearance > 50 mL/min and 7.5 mg/kg when creatinine clearance is 30–50 mL/min [130]. Telavancin is not indicated if creatinine clearance < 30 mL/min [130]. It has a plasma protein binding of 90% and a volume of distribution in steady state of 10.878 L [130]. The ratio of the area under the plasma concentration-time curve from time zero to 24 h of unbound plasma concentrations (AUC_24_) to the MIC_90_ of *S. aureus* and *S. epidermidis* is enough to achieve pharmacokinetic/pharmacodynamic (pK/pD) targets for optimal bacterial killing [130]. Despite further investigations required to determine the penetration of telavancin into ELF, the AUC_24_/MIC_90_ ratio for *S. aureus* suggests that bactericidal activity in ELF is expected [130]. A phase I, randomised, double-blind, placebo-controlled crossover study found that telavancin at intended therapeutic doses did not affect the pharmacokinetics of intravenous midazolam, a P450 CYP3A probe substrate, concluding that telavancin is unlikely to inhibit hepatic CYP3A activity [143]. Therefore, dose adjustment is not recommended for patients with mild to moderate hepatic impairment (Child-Pugh B), however caution should be exercises for severe hepatic impairment (Child-Pugh C) due to lack of available data [130]. Drug interactions have not been reported with aztreonam and piperacillin/tazobactam and can be safely co-administered. Moreover, enzyme inducers/inhibitors do not seem to affect its metabolism [144,145].

Two methodologically, identical phase III, randomised, non-inferiority, interventional clinical trial (NCT00107952 and NCT00124020), ATTAIN1 and ATTAIN2, comparing the safety and effectiveness of intravenous telavancin and vancomycin for the treatment of HAP, specifically due to MRSA, were conducted on 1503 participants [146]. Telavancin achieved numerically higher cure rates than vancomycin (82.4% vs. 80.74%), however this was not statistically significant [147]. In patients with VAP, telavancin also produced a higher but statistically insignificant cure rate compared to vancomycin (80.3% vs. 67.6%). In a patient population with HAP/VAP with at least one Gram-positive pathogen who also had concurrent *S. aureus* bacteraemia, those treated with telavancin had a 28-day all-cause mortality rate of 40% compared with 39.5% for vancomycin-treated patients [131]. A decision-analytic model found telavancin for monomicrobial *S. aureus* HAP was associated with higher drug (USD 2082) and nephrotoxicity (USD 467) costs, but lower ICU (-USD 1738) and ventilator (-USD 114) costs, resulting in a net cost saving of USD 907 per patient, compared to vancomycin [148]. Serious adverse events were reported in 34.14% (127/372) including respiratory failure (14/372, 3.76%), septic shock (13/372, 3.49%), multi-organ failure (11/372, 2.96%) and acute renal failure (11/372, 2.96%) [146]. Non-serious adverse events of telavancin were reported in 86.29% (321/372), including constipation (8.60%), anaemia (8.06%), hypokalaemia (8.06%), nausea (7.26%) and vomiting (5.65%) [146]. Compared to vancomycin, the percentage of patients on telavancin reporting at least one treatment-emergent adverse event was similar (80% vs. 79%) [147]. Similarly to vancomycin, rapid infusion may result in “red-man syndrome”-like reaction so intravenous infusion of telavancin needs to be over 60 min [145]. Finally, it should be noted that telavancin is indicated for the treatment of HAP/VAP caused by *S. aureus*, both methicillin-susceptible and -resistant isolates, only when alternative treatments are not suitable [142].

### 3.3. Ceftolozane/Tazobactam

Ceftolozane/tazobactam, brand name Zerbaxa™, is a novel broad-spectrum fifth generation cephalosporin combined with a β-lactamase inhibitor marketed by Merck & Co. (Kenilworth, New Jersey, United States) in the United States and Canada and Merck Sharp & Dohme (MSD) elsewhere [149,150]. It was initially approved by the FDA in December 2014 and the EMA in September 2015 for the treatment of complicated intra-abdominal infections (cIAI) and complicated urinary tract infections (cUTI), inclusive of acute pyelonephritis. Ceftolozane/tazobactam received a post-authorisation approval by the FDA in June 2019 and the EMA in July 2019 for the treatment of bacterial HAP and VAP [149,150].

The mechanism of action of ceftolozane, similar to other β-lactam antibiotics, depends on a chemical reaction with PBPs from bacteria, forming stable, inactive acyl-enzymes [151]. This inhibits transpeptidase activity of PBPs, which prevents further cross-linking of peptidoglycan in the bacterial cell wall, weakening its structure and ultimately causing lysis due to osmotic imbalance [151]. Tazobactam is an irreversible inhibitor of most class A β-lactamases, including ESBLs, and some class C β-lactamases of the Ambler classification scheme. Its mechanism of action depends on a chemical reaction with the β-lactamase active site, forming a stable imine acyl-enzyme complex [151].

From a chemical perspective, ceftolozane is a semi-synthetic broad-spectrum cephalosporin, structurally related to ceftazidime [151]. Similar to ceftazidime and other broad-spectrum cephalosporins, ceftolozane contains an aminothiadiazole ring on its 7-position side-chain, which provides enhanced activity against Gram-negative bacteria; an oxime group, which confers stability against β-lactamases; and a dimethylacetic acid moiety, which improves antipseudomonal activity [151]. A structural detail is responsible for the differential activity of ceftolozane: a pyrazole ring on its 3-position side-chain, which prevents hydrolysis and improves stability against AmpC β-lactamase-overproducing *P. aeruginosa* [151]. A 2-aminoethylureido group on the 4-position side-chain of the pyrazole ring grants activity against this microorganism and a weaker convulsing-inducing effect than that of ceftazidime or cefepime [152,153].

Ceftolozane/tazobactam exhibits enhanced antipseudomonal activity, with an excellent in vitro activity against PBP1b, 1c, 2 and 3 of multi-drug resistant (MDR) and extensively drug-resistant (XDR) *P. aeruginosa*, including carbapenem-resistant organisms with porin loss, up-regulated efflux pumps and derepressed AmpC mechanisms [154]. The MIC of ceftolozane against *P. aeruginosa* is 8- to 16-fold lower than that of ceftazidime, imipenem or ciprofloxacin [152]. Data from the SMART surveillance program in United states showed that overall susceptibility of *P. aeruginosa* isolates was higher for ceftolozane/tazobactam compared to ceftazidime, meropenem, piperacillin/tazobactam and levofloxacin (94.7% versus 76.8% versus 77.0% versus 70.2% versus 69.0%, respectively) [155].

Ceftolozane/tazobactam exhibits great activity against *Enterobacteriaceae* (*E. coli*, *Klebsiella*, *Enterobacter*, *Proteus*, *Salmonella* and *Serratia)*, including ESBL-producing strains, but shows no activity against carbapenemases (except some OXA-48-producing microorganisms) and plasmid-determined AmpC β-lactamases [149,150]. *Haemophilus*, *Moraxella*, *Burkholderia pseudomallei* and about 50% of *Burkholderia cepacia* and *Chryseobacterium* are susceptible [112,154,156,157]. Most *S. maltophilia*, *Achromobacter* and *Acinetobacter* isolates are resistant [112,113,156]. It is active against *Streptococcus* spp. (MIC_90_ 0.5 mg/L) and moderately active against *S. pneumoniae* (MIC_90_ 4 mg/L), whereas *S. aureus* and *Enterococcus* spp. are non-susceptible [112,158]. This antibiotic is also active against some anaerobes, including *Fusobacterium*, *Prevotella*, *Cutibacterium acnes*, *Clostridium perfringens* and *Bacteroides fragilis* [112,159].

The main mechanism of acquired resistance to ceftolozane/tazobactam in clinical isolates of *P. aeruginosa* is the presence of β-lactamases that hydrolyseceftolozane and are not inhibited by tazobactam (i.e., metallo-β-lactamases, OXA-type ESBLs or GES-type enzymes) [49,154,160,161]. Another possible mechanism of resistance are mutations in the resident AmpC β-lactamase, leading to overexpression, which could be responsible for increased MICs following clinical use [162,163,164,165]. However, ceftolozane/tazobactam tends to select resistant mutants with a lower frequency than other antipseudomonal agents (meropenem, ceftazidime or ciprofloxacin) [154,162]. Production of carbapenemases is the major mechanism of resistance to ceftolozane/tazobactam in *Enterobacteriaceae* and most carbapenemase-producing *Enterobacteriaceae* (CPE) are usually non-susceptible to this antibiotic [154].

Ceftolozane/tazobactam demonstrated dose-independent linear kinetics [150]. Pharmacokinetic parameters: C_max_ 69.1/18.4 mg/L; time until C_max_ is reached (t_max_) 1 h; area under the concentration-time curve (AUC) 172/24.4 mg × h/L; plasma protein binding 16–21/30%; volume of distribution (Vd) 13.5/18.2 L; no appreciable metabolism and no interactions with any OAT1/OAT3, CYP1A2 and CYP3A4 substrates that were tested; plasma elimination half-life (t_1/2_) 2.6 h; excretion > 95/> 80% unchanged in urine [149,150,166]. As for all cephalosporins, the pK/pD index that best relates to therapeutic success is the fraction of time during the dosing interval for which the free drug concentration remains above the MIC for the infecting microorganism (%*fT* > MIC) [167]. A phase I study assessing intrapulmonary penetration of ceftolozane/tazobactam after a 1 h infusion of a 1.5 g dose in healthy subjects demonstrated both ceftolozane and tazobactam penetrated well into the pulmonary ELF/plasma ratios 48% and 44%, respectively, and ceftolozane ELF concentrations exceeded 8 mg/L for >60% of the 8-h dosing interval [168]. The pivotal study leading to the approval of ceftolozane/tazobactam for the indication of bacterial HAP and VAP, ASPECT-NP (NCT02070757) [169], used a high dose regimen of the antibiotic to ensure therapeutic drug concentration at the site of infection, given the complex pK/pD interactions in critically ill patients, so that it covered pathogens with higher MICs likely causative of bacterial HAP/VAP [170]. Ceftolozane/tazobactam demonstrated higher stability against emergence of non-susceptibility during treatment, compared to meropenem (0% vs. 22%) [169]. Among *P. aeruginosa* isolates that developed non-susceptibility to meropenem, none developed co-resistance to ceftolozane/tazobactam [169]. A more recent phase I study assessed lung penetration of ceftolozane/tazobactam with this higher dose regimen (2 g/1 g, every 8 h) in mechanically ventilated patients with pneumonia, with ELF/plasma ratios 50% and 62%, respectively, and mean ELF concentrations > 4 mg/L for ceftolozane and >1 mg/L for tazobactam for 100% of the dosing interval [171].

The approved dosing regimen of ceftolozane/tazobactam for adults with HAP/VAP is 3 g (2 g ceftolozane/1 g tazobactam) every 8 h by IV 1-h infusion, for 8–14 days [149,150,169]. Whereas no change is required in patients with liver failure, dosage adjustment is required in patients with impaired renal function, by reducing the dose and keeping the dosing interval: 1.5 g/8 h for patients with eGFR 30–50 mL/min; 0.75 g/8 h for patients with eGFR 15–30 mL/min; single 2.25 g loading dose followed by 0.45 g/8 h for patients with eGFR < 15 mL/min or on chronic haemodialysis (60% of the dose is removed by dialysis) [149,150]. Although dosing in patients under CRRT is not well stablished, a dose of 3 g/8 h seems to be safe and involves less risk of clinical failure when compared to lower dose regimens [172,173,174]. A recent ex vivo and in vivo study concluded that the influence of ECMO on the pharmacokinetics of ceftolozane/tazobactam is not clinically relevant, so standard doses should be effective for the treatment of these patients [175]. A case report in a lung transplant recipient on ECMO found similar *invivo* results [176].

The approval of ceftolozane/tazobactam for the indication of bacterial HAP and VAP was based on a randomised, controlled, double-blind, phase III, non-inferiority trial study in 726 adult patients hospitalised with bacterial HAP/VAP (NCT02070757) [169]. This study compared ceftolozane/tazobactam to meropenem for 8–14 days of therapy [169]. Efficacy was assessed based on all-cause mortality at day 28 (24.0% for ceftolozane/tazobactam vs. 25.3% for meropenem) and clinical cure, defined as complete resolution or significant improvement in signs and symptoms of the index infection at the test-of-cure visit which occurred 7 to 14 days after the end of treatment (54.4% for ceftolozane/tazobactam vs. 53.3% for meropenem) [169].

Although ceftolozane/tazobactam does not have any clinically relevant interaction with other drugs, it presents Y-site administration incompatibility with albumin, amphotericin B, caspofungin, ciclosporin, nicardipine, phenytoin and propofol [112].

Most common side effects of ceftolozane/tazobactam are mild and include gastrointestinal symptoms like nausea (7.9%), vomiting (1–3.3%), diarrhoea (1–6.2%) or abdominal pain (1.2%), alteration of liver function tests (3%), pyrexia (5.2%), hypokalaemia (2.9%) and thrombocytosis (1.9%) [149,150,169,177]. The development of a positive direct antiglobulin test (Coombs test) may occur during treatment, with no evidence of associated haemolysis in clinical trials [149,150]. *C. difficile* colitis cases have been reported with ceftolozane/tazobactam (1%) [149,150,169].

Ceftolozane/tazobactam represents a promising addition to the available antibacterial armamentarium because of its efficacy for the treatment of Gram-negative NP, especially when caused by *Enterobacteriaceae* (including ESBL producers) or *P. aeruginosa* (including MDR and XDR isolates).

### 3.4. Ceftazidime/Avibactam

Ceftazidime/avibactam, marketed by Allergan Inc. (Irvine, CA, U.S.) in US and Canada under the trade name Avycaz™, and by Pfizer (Manhattan, NY, US) in the rest of the world under the trade name Zavicefta™, is a combination of a 3rd-generation cephalosporin (ceftazidime) and a novel non-β-lactam β-lactamase inhibitor (avibactam) [178,179]. The combination is approved by the FDA in 2015 and the EMA in 2016 for the treatment of cIAI (in combination with metronidazole), cUTI (including pyelonephritis), bacterial HAP and VAP, bacteraemia related to any of the previous infections, and infections due to aerobic Gram-negative organisms in patients with limited treatment options [178,179].

Ceftazidime is a well-known, 3rd-generation, broad spectrum cephalosporin which exerts its action by penetrating the cell wall of bacteria and binding to PBP targets [180]. As with other β-lactam antibiotics, this leads to inhibition of peptidoglycan crosslinking during cell wall synthesis, ultimately leading to lysis and death of bacterial cells [180,181]. Avibactam is a first-in-class non-β-lactam β-lactamase inhibitor, without intrinsic antimicrobial activity [182]. When used in combination with a β-lactam, avibactam protects it from degradation by a wide range of β-lactamases [182,183,184]. Avibactam binds covalently to β-lactamases in a reversible way by a process involving acylation and deacylation, without hydrolysis, which constitutes one of the differential features of this molecule when compared to β-lactam β-lactamase inhibitors [182,183,184,185,186]. Avibactam effectively inhibits serine β-lactamases of Ambler class A (CTX-M, SHV, TEM, KPC, GES, PER and SME), chromosomally mediated (AmpC) and plasmid-mediated (FOX, MOX, CMY, LAT, ACC and DHA) class C β-lactamases, and some class D β-lactamases (especially OXA-48, and OXA-2, OXA-5/10 and OXA-50 to a lesser extent) [167,168,169]. However, avibactam does not show any activity against metallo-β-lactamases [187,188,189,190].

Avibactam does not improve the limited antimicrobial spectrum of activity of ceftazidime against Gram-positive bacteria and anaerobes [181]. The combination is active against *Enterobacteriaceae*, including ESBL-, carbapenemase- (KPC, GES and OXA-48) and AmpC-producing isolates [181]. A recent surveillance study showed that more than 99% of *Enterobacteriaceae* isolates were susceptible to ceftazidime/avibactam, with a MIC_90_ 0.5 µg/mL, seven doubling dilutions lower than the MIC_90_ for ceftazidime alone (64 µg/mL) [191]. While most ceftazidime-avibactam nonsusceptible *Enterobacteriaceae* isolates were also carbapenem-resistant (>95%) [191], a significant proportion of meropenem-nonsusceptible *Enterobacteriaceae* were susceptible to ceftazidime/avibactam (>80%) [188]. Ceftazidime/avibactam shows excellent activity against *P. aeruginosa*, with >90% of isolates susceptible, with a MIC_90_ 8 µg/mL, three doubling dilutions lower than the MIC_90_ for ceftazidime alone (64 µg/mL) [192]. Avibactam is able to restore ceftazidime activity in more than two thirds of the ceftazidime-non-susceptible isolates of *P. aeruginosa* [192]. Almost three out of four carbapenem-nonsusceptible or KPC-producing isolates of *P. aeruginosa* are susceptible to ceftazidime/avibactam [192,193]. Ceftazidime/avibactam is active against *B. cepacia* complex and *Burkholderia gladioli* isolates which are nonsusceptible to ceftazidime, as they usually produce PenA and PenI β-lactamases (class A), which are structurally related to KPC and, thus, are inhibited by avibactam [112]. Activity of avibactam against *Burkholderia* spp. can be improved by association of piperacillin [112,194]. Nonsusceptibility of Acinetobacter to ceftazidime is not modified by the combination with avibactam [112,195]. As *S. maltophilia*, *Elizabethkingia meningoseptica* and *Aeromonas* spp. produce a chromosomal metallo-β-lactamase which is not inhibited by avibactam; they are resistant to ceftazidime/avibactam [112]. Compared to ceftolozane/tazobactam, ceftazidime/avibactam has lower MICs against ESBL-producing and better in vitro activity against carbapenem-resistant *Enterobacteriaceae* (CRE), while exhibiting higher MICs against *P. aeruginosa* [196,197]. Even though, susceptibility rates of both agents are similar [196,197].

Emergence of resistance to ceftazidime/avibactam appears to be low [198,199]. A recent surveillance study showed 0.5% of *Enterobacteriaceae* isolates and 8% of *P. aeruginosa* isolates were non-susceptible to ceftazidime/avibactam [191,192]. A frequent mechanism of resistance in clinical practice is the presence of β-lactamases that are not efficiently inhibited by avibactam [181]. *Klebsiella* isolates with mutations in porins OmpK36 and OmpK35 or overexpressing mexAB-OprM are less susceptible to ceftazidime/avibactam [112,200,201]. Treatment of infections caused by KPC-2 or KPC-3 producing strains might lead to selection of resistance to avibactam [202,203], usually associated with restoration of susceptibility to meropenem by loss of the carbapenemase activity and an increased ceftazidimase activity [204,205]. Therefore, association of a carbapenem has been suggested as a strategy to prevent the development of ceftazidime/avibactam resistance in KPC-producing *Enterobacteriaceae* [206]. Other potential mechanisms of resistance are mutant or acquired PBPs, decreased outer membrane permeability and active efflux pumps [178,179].

Ceftazidime and avibactam both have a linear pharmacokinetic profile across the studied dose range [178,179]. Pharmacokinetic parameters after a 2-h IV infusion of a 2 g/0.5 g dose of ceftazidime/avibactam in healthy adults: C_max_ 88/15 mg/L; area under the concentration-time curve (AUC) 289/42 mg × h/L; plasma protein binding 8/8%; volume of distribution (Vd) 17/22.2 L; none of the compounds undergo significant metabolism; plasma elimination half-life (t_1/2_) 3.3/2.2 h; both ceftazidime and avibactam are eliminated via the kidneys (>80% of ceftazidime and >95% of avibactam unchanged) [178,179]. The pK/pD index that best relates to therapeutic success is the fraction of time during the dosing interval for which the free avibactam concentration remains above the threshold concentration (%*fT* > TC) [112]. Threshold concentration (TC) is defined as the minimal concentration of the inhibitor that is able to restore the activity of the β-lactam antibiotic (TC = 0.5 mg/L for and TC = 1 mg/L for *P. aeruginosa*, when avibactam is combined with ceftazidime) [112]. Penetration of ceftazidime/avibactam into the ELF in healthy subjects has been assessed in a phase I study [207]. This study found that plasma and ELF concentrations increase in a dose-dependent manner for both drugs, with a plasma/ELF ratio close to 30% for both drugs [207].

The approved dosing regimen of ceftazidime/avibactam for adults with bacterial HAP/VAP is 2.5 g (2 g ceftazidime/0.5 g avibactam) every 8 h by IV 2-h infusion, for 7–14 days [178,179]. While no dosage change is required in patients with deranged liver function, patients with renal failure should receive lower doses, as both compounds are mainly eliminated via the kidneys: 1 g/0.25 g every 8 h for patients with eGFR 30–50 mL/min; 0.75 g/0.1875 g every 12 h for patients with eGFR 15–30 mL/min, every 24 h for patients with eGFR 5–15 mL/h and every 48 h for patients with end stage renal disease or on haemodialysis (80% of the dose is removed by dialysis) [178,179]. Dosing in patients under CRRT remains controversial, as it has been proposed according to case reports, from 1 g/0.25 g every 12 h to standard doses (2 g/1 g every 8 h) [208,209,210]. Pharmacokinetics of ceftazidime/avibactam has not been assessed in patients under ECMO support.

The approval of ceftazidime/avibactam for the indication of bacterial HAP and VAP was based on a randomised, controlled, double-blind, phase III non-inferiority study in 879 adult patients with NP, including bacterial VAP (NCT01808092) [211]. This study compared ceftazidime/avibactam (2 g/0.5 g infused over 2 h, every 8 h, for 7–14 days) to meropenem (1 g infused over 30 min, every 8 h, for 7–14 days) [211]. Efficacy was assessed based on clinical cure at the test-of-cure visit (21–25 days after randomisation [211]. Based on this, ceftazidime/avibactam was not inferior to meropenem for the treatment of bacterial HAP/VAP (68.8% vs. 73.0%, *p* = 0.007, in the clinically modified intention-to-treat population; and 77.4% vs. 78.1%, *p* = 0.001, in the clinically evaluable population) [211]. This study remarks the potential of ceftazidime/avibactam to be used as a carbapenem-sparing agent for the treatment of bacterial HAP/VAP caused by Gram-negative microorganisms [211].

Ceftazidime/avibactam does not inhibit any major renal or hepatic transporters in the clinical setting, so interactions via these mechanisms are not expected [178,179]. Avibactam is a substrate of OAT1 and OAT3, so OAT inhibitors (i.e., probenecid) might alter its elimination [178,179]. Association of ceftazidime/avibactam with aztreonam, meropenem, amikacin and fosfomycin are synergistic [112,212].

Common side effects are usually mild and include gastrointestinal symptoms like diarrhoea (15%), vomiting (6%), constipation (6%), nausea (3%) or abdominal pain (2%), alteration of complete blood count (eosinophilia, thrombocytosis, thrombocytopenia) (<1%), alteration of liver function tests (4%), headache (3%), hypokalaemia (11%), pyrexia (2%), candidiasis (<1%) and infusion site reactions [178,179,211]. The development of the Coombs test may occur during treatment, with no evidence of associated haemolysis in clinical trials [178]. *C. difficile* colitis cases have been reported on patients under treatment with ceftazidime/avibactam [178,179]

Ceftazidime/avibactam is an excellent alternative for the treatment of bacterial HAP/VAP caused by Gram-negative microorganisms (i.e., *Enterobacteriaceae* and *Pseudomonas*), including certain carbapenemase-producing isolates.

### 3.5. Meropenem/Vaborbactam

Meropenem/vaborbactam, trade name Vabomere™, marketed by Melinta Therapeutics Inc. (Morristown, NJ, US) in US and by Menarini Group (Florence, Tuscany, Italy) in the rest of the world, is a combination of a broad-spectrum carbapenem (meropenem) and a novel cyclic boronic acid-based β-lactamase inhibitor (vaborbactam) [213,214]. The combination was first approved by the FDA in 2017 for the treatment of cUTI and by the EMA in 2018 for the treatment of cUTI including pyelonephritis, cIAI and bacterial HAP and VAP [213,214].

Meropenem is a group 2 carbapenem which exerts its action by penetrating the cell wall of a wide range of Gram- and Gram-negative bacteria, to reach PBP targets [215]. It shows great affinity to PBP-2, -3 and -4 of *E. coli* and *P. aeruginosa*, and to PBP-1, -2 and -4 of *S. aureus* [215,216]. Although meropenem is stable to hydrolysis by penicillinases and cephalosporinases, the increasing prevalence of carbapenemases threatens the clinical use of this family of antibiotics [215]. The use of a β-lactam and β-lactamase inhibitor combination is an effective strategy to overcome this type of resistance [217]. Vaborbactam, previously known as RPX7009, is a novel β-lactamase inhibitor based on a cyclic boronic acid pharmacophore [217]. Vaborbactam enters the periplasmic space of *K. pneumoniae* using porins OmpK36 (preferred) and OmpK35 [218]. It potently inhibits serine β-lactamases of class A (CTX-M, SHV, TEM, and carbapenemases KPC, BKC-1 and FRI-1) and class C (AmpC, FOX, P99 and MIR), by forming reversible covalent bonds not leading to hydrolysis [112,217]. However, it does not show any activity against class B (metallo-β-lactamases) and class D (OXA-48) β-lactamases [217].

Vaborbactam does not improve the antimicrobial spectrum of activity of meropenem against Gram-positive bacteria, anaerobes, *P. aeruginosa* and *Acinetobacter* spp. [219,220]. The combination is active against *Enterobacteriaceae*, including ESBL-, cephamycinase- and class A serine carbapenemases-producing isolates [215]. Meropenem/vaborbactam retains activity against KPC-producing microorganisms with mutations which lead to resistance to ceftazidime/avibactam (i.e., KPC-8, KPC-31) [221]. Resistance was reported in *Stenotrophomonas*, *Elizabethkingia* and *Aeromonas* [112,215].

Emergence of resistance to meropenem/vaborbactam can be due to loss of outer membrane porins OmpK36 and OmpK35 in *K. pneumoniae*, as it has been demonstrated in in vitro experiments [217]. Decreased expression of porins OmpC and OmpF has also been described to cause resistance to meropenem/vaborbactam in Enterobacter [112]. Overexpression of the multidrug efflux pump AcrAB-ToIC is associated with reduced susceptibility of *Enterobacteriaceae* [217]. KPC-producing isolates lacking both porins and overexpressing AcrAB-ToIC significantly reduce susceptibility to meropenem/vaborbactam, although meropenem MICs were normally <8 mg/L with varborbactam concentrations of 8 mg/L [217]. Overexpression of KPC due to increased blaKPC gene copy number has also been described to reduce susceptibility to meropenem/vaborbactam [218].

Pharmacokinetic parameters after a 3-h IV infusion of a 2 g/2 g dose of meropenem/vaborbactam in healthy adults: C_max_ 46/50 mg/L; area under the concentration-time curve (AUC) 142/168 mg × h/L; plasma protein binding 2/33%; volume of distribution (Vd) 21.5/21.5 L; 20–30% of meropenem undergoes metabolism whereas there is no significant metabolism of vaborbactam; plasma elimination half-life (t_1/2_) 1.5/2 h; both meropenem and vaborbactam are eliminated via the kidneys (90% of vaborbactam unchanged) [213,214,222]. These parameters have also been studied in phase III studies with similar results [223]. The pK/pD index that best relates to therapeutic success is the fraction of time during the dosing interval for which the free vaborbactam concentration remains above the threshold concentration ((*%fT* > TC)) [112]. TC is defined as the minimal concentration of the inhibitor that is able to restore the activity of the β-lactam antibiotic (TC = 8 mg/L for *Enterobacteriaceae*, when vaborbactam is combined with meropenem) [112]. The pD parameter associated with suppression of resistance is fAUC (AUC over the first 24h of treatment) of vaborbactam/MIC > 24 [112]. Penetration of meropenem/vaborbactam into the ELF in healthy subjects has been assessed in a phase I study [224]. When unbound plasma concentrations were considered, the ratios of ELF to total plasma meropenem and vaborbactam concentrations were 65% and 79%, respectively [224].

The approved dosing regimen of meropenem/vaborbactam for adults with bacterial HAP/VAP is 4 g (2 g meropenem/2 g vaborbactam) every 8 h by IV 3-h infusion, for 7–14 days [213,214]. While no dosage change is required in patients with liver function impairment, patients with renal failure should receive lower doses, as both compounds are mainly eliminated via the kidneys: 2 g/8 h for patients with estimated glomerular filtration rate (eGFR) 30–50 mL/min; 2 g/12 h for patients with eGFR 15–30 mL/min; 1 g/12 h for patients with eGFR < 15 mL/min or on chronic haemodialysis (>50% of the dose is removed by dialysis) [213,214]. Dosing in patients under CRRT has been proposed according to data from an ex vivo study: 1 g/8 h for low effluent flow rates (1–2 L/h) and 2 g/8 h for high effluent flow rates (3–4 L/h) [225]. Pharmacokinetics of meropenem/vaborbactam has not been studied in patients receiving extracorporeal membrane oxygenation (ECMO) support.

The approval of meropenem/vaborbactam for the indication of bacterial HAP and VAP was based on a randomised, controlled, open-label, phase III study in 77 adult patients with cUTI, cIAI, bacterial HAP/VAP or bacteraemia suspected or documented to be caused by CRE (NCT02168946) [226]. This study compared meropenem/vaborbactam (4 g infused over 3 h, every 8 h, for 7–14 days) with the best available antibiotic treatment (polymyxin, carbapenem, aminoglycoside or tigecycline, alone or in combination; or ceftazidime/avibactam alone) [226]. Efficacy was assessed based on all-cause mortality at day 28 (22.2 vs. 44.4%, *p* = 0.25, in patients with bacterial HAP/VAP or bacteraemia) and also, in terms of clinical and microbiologic cure, and overall success [226]. The study concludes that use of meropenem/vaborbactam for CRE infections is associated with better results compared to the best available therapy [226]. However, some methodological issues of this study might limit the interpretation of the results [226].

The most relevant clinical interaction of meropenem is the reduction of plasma concentration of valproate [213,214,227]. In vitro data suggest that vaborbactam may inhibit CYP2D6, so patients receiving treatment with CYP2D6 substrates with narrow therapeutic index (i.e., dextromethorphan, desipramine, venlafaxine or metoprolol) should be monitored for signs of toxicity [213]. Meropenem/vaborbactam presents Y-site administration incompatibility with albumin, amiodarone, anidulafungin, calcium chloride, caspofungin, ceftaroline, ciprofloxacin, daptomycin, diphenhydramine, dobutamine, isavuconazole, midazolam, ondansetron and phenytoin [112,213,214].

Common side effects reported in clinical trials were headache (8.8%), diarrhoea (3.3–12%), infusion site phlebitis (4.4%) and nausea (1.8%) [213,214,226,228]. Other frequent adverse effects are hypotension (8%), alteration of liver function tests (1.8%), pyrexia (1.5%), hypokalaemia (1.1–10%), hypoglycaemia (<1%) and thrombocytosis (<1%) [213,214].

Meropenem/vaborbactam represents a promising alternative for the treatment of difficult to treat *Enterobacteriaceae*, including ESBL-, cephamycinase- and class A serine carbapenemases-producing isolates [229]. However, it provides no added antipseudomonal coverage when compared to meropenem alone, and it poses logistical challenges as it needs to be administered over 3 h due to rapid irreversibility of the β-lactamase inhibitor [229].

The summary of the dosage and suggested treatment duration of the novel antibiotics for NP, as well as other approved indications, are summarised in Table 3, while their main spectrum of activity is briefly depicted in Table 4. For details on the overall management of NP, including when to start and how to select the initial empirical antibiotic regimen, when/how to re-assess the initial antibiotics and when to discontinue the antibiotic treatment, we refer the reader to relevant guidelines [1,230].

## 4. Conclusions

Although the prompt diagnosis and appropriate treatment of NP is essential for the improvement of outcomes, to date, there is no diagnostic gold standard. CXR along with the clinical symptoms and signs still guide the diagnosis, however, it has been shown by several studies that CXR has both low sensitivity and specificity. The use of LUS and LRCT has been increasing during the last decade. LUS is simple, fast, can be easily repeated to allow follow up at the point-of-care and represents a very promising adjunct imaging tool for NP diagnosis. LRCT and ultra LRCT have higher sensitivity than CXR with almost similar radiation exposure, giving a great advantage in their use and the potential to become valuable assets in NP diagnosis. Notwithstanding, for both LUS and LRCT/ultra LRCT, the utility of these imaging modalities needs to be validated in well-designed large-scale NP trials.

The advances in rapid molecular techniques for microbiological confirmation, such as rapid syndromic multiplex PCR tests have enhanced the diagnostic armamentarium of NP. They are impressively faster than traditional cultures, significantly reducing the time from sampling to pathogens identification, as well as providing information for several resistance markers. They have the potential to change the scene of the diagnostic and management approach of NP in the future, improving outcomes, as well as contributing to better antibiotic stewardship. However, their role for guiding early targeted therapy, de-escalation, and their cost effectiveness needs to be further evaluated in large, well-designed studies, therefore, they are not yet included in international guidelines for NP diagnosis. VOC and eNose, on the other hand, coupled with artificial intelligence, is a positive development in the medical field for the non-invasive and prompt diagnosis of NP. Nevertheless, its potential is not yet fully recognised and further research into this field is required in order to be applied in clinical practice.

With reference to NP management, a small number of antibiotics received approval for the indication of NP during the last decade. Ceftobiprole medocaril is the first anti-MRSA cephalosporin that has been approved for NP (excluding VAP). Telavancin, a semi-synthetic lipoglycopeptide derivative of vancomycin with enhanced activity and reduced resistance potential, has been approved for HAP/VAP caused by *S. aureus,* including bacteraemic cases. Ceftolozane/tazobactam, ceftazidime/avibactam and meropenem/vaborbactam are novel antibiotics (beta-lactam/beta-lactamase inhibitor) approved for HAP and VAP, with good results for several Gram-negative bacilli that represent a major problem, especially in the critical care setting. However, of note, they are not active against MBL producers and carbapenem-resistant *A. baumannii.* It should be emphasised that continuous surveillance is very important to monitor resistance development to these newer antibiotics for NP, as well as judicious use, in order to increase their “self life”.

## Figures and Tables

**Table 1:** Pathogens and AMR genes detected, suitable types of samples and time to results of commercially available multiplex PCR panels used for NP.

Multiplex PCR Panel	Type of Sample and Time to Results	Performance ^§^	Pathogens/Markers of Resistance Genes Detected	
BioFire^®^ FilmArray^®^ Pneumonia Panel and Pneumonia Panel Plus (bioMérieux SA, France) [https://www.biofiredx.com/products/the-filmarray-panels/filmarray-pneumonia; Access date 20 February 2021, https://www.biomerieux-diagnostics.com/biofire-filmarray-pneumonia-panel; Access date 20 February 2021]	BAL/mini-BAL, tracheal aspirate, induced and expectorated sputum Time to results: 1 h	Both panels: BAL/BAL-like: Sens/Spec= 96.2%/98.3% Sputum: Sens/Spec= 96.3%/97.2%	Bacteria *Acinetobacter calcoaceticus-baumannii complex* *Enterobacter cloacae* *Escherichia coli* *Haemophilus influenzae* *Klebsiella aerogenes* *Klebsiella oxytoca* *Klebsiella pneumoniae group* *Moraxella catarrhalis* *Proteus* spp. *Pseudomonas aeruginosa* *Serratia marcescens* *Staphylococcus aureus* *Streptococcus agalactiae* *Streptococcus pneumoniae* *Streptococcus pyogenes* *Legionella pneumophila* *Mycoplasma pneumoniae* *Chlamydia pneumoniae*	Viruses Influenza A and B Adenovirus Coronavirus Parainfluenza virus Respiratory Syncytial virus Human Rhinovirus/Enterovirus Human Metapneumovirus Middle East Respiratory Syndrome Coronavirus (MERS-CoV) * Antibiotic resistance genes CTX-M, KPC, NDM Oxa48-like, VIM, IMP, mecA/mecC and MREJ
Unyvero Lower Respiratory Tract (LRT) Panel and LRT BAL (Curetis AG, USA) ** [https://www.curetisusa.com/wp-content/uploads/Unyvero-Pneumonia-Panel-Flyer-PN3677A.pdf; Access date 20 February 2021]	BAL/mini-BAL or tracheal aspirate Time to results: 5 h	Both panels: Sens/Spec = 91.4%/99.5%	Bacteria *Acinetobacter* spp. *Chlamydia pneumoniae* *Citrobacter freundii* *Enterobacter cloacae complex* *Escherichia coli* *Haemophilus influenzae* *Klebsiella oxytoca* *Klebsiella pneumoniae* *Klebsiella variicola* *Legionella pneumophila* *Moraxella catarrhalis* *Morganella morganii* *Mycoplasma pneumoniae* *Proteus* spp. *Pseudomonas aeruginosa* *Serratia marcescens* *Staphylococcus aureus Stenotrophomonas maltophilia* *Streptococcus pneumoniae*	Other/Fungi *** Pneumocystis jirovecii Antibiotic resistance genes KPC, NDM, OXA-23, OXA-24, OXA-48, OXA-58, VIM, CTX-M, mecA, TEM
Unyvero Hospitalised Pneumonia (HPN) Cartridge (Curetis AG, USA) [https://www.curetisusa.com/wp-content/uploads/Unyvero-LRT-Pneumonia-Brochure.pdf; Access date 20 February 2021]	BAL/mini-BAL, tracheal aspirate, sputum Time to results: 4–5 h	For microorganisms: Sens/Spec= 92.5%/97.4% For AMR markers Sens/Spec = 93%/98.8%	Bacteria Same as Unyvero LRT BAL Panel and additionally *Chlamydophila pneumoniae*	Other/Fungi *Pneumocystis jirovecii* Antibiotic resistance genes ERMB, mecA/mecC, TEM SHV, CTX-M, KPC, NDM, OXA-23, OXA-24/40, OXA-48, OXA-58, VIM, SUL1, gyrA83, gyrA87
Unyvero P55 panel (Curetis AG, USA) [https://curetis.com/wp-content/uploads/20150416_Curetis_P55_study_completion_EN_FINAL_APPROVED.pdf;Access date 20 February 2021]	BAL/mini-BAL, tracheal aspirate, sputum Time to results: 4–5 h	Sens/Spec= 94%/99.4%	Bacteria Same as Unyvero LRT BAL Panel and additionally: *Klebsiella aerogenes* (previously known as *Enterobacter aerogenes*)	Other/Fungi *Pneumocystis jirovecii* Antibiotic resistance genes ERMB, mecA/mecC, TEM SHV, CTX-M, IMP, KPC, NDM, OXA-23, OXA-24, OXA-48, OXA-58, VIM, SUL1, gyrA83 gyrA87

§ Performance provided by the respective manufacturers; * MERS-CoV is only available in the Pneumonia Panel plus; ** Unyvero LRT panel is used in tracheal aspirates and Unyvero LRT BAL panel is used in BAL and mini-BAL samples; *** *Pneumocystis jirovecii* is only available in Unyvero LRT BAL panel; AMR: Antimicrobial resistance, BAL: Bronchoalveolar lavage fluid, Sens: Sensitivity, Spec: Specificity, spp: Species.

**Table 2:** Advantages and disadvantages of multiplex PCR panels.

Advantages	Disadvantages
Exceptionally faster time to results for pathogen and resistance profiles: major utility for prompt treatment modification and effective patient management	Over-detection of microbial and viral genome: problem in results interpretation: pathogen or coloniser? (may be partially solved with semi-quantification of bacterial targets)
Multiple targets detection at the same and Detection of viral and atypical pathogens as well	The presence of a resistance gene marker may not be linked to the detected microorganism, but to other co-existent organisms either undetectable or below the detection limit, thus making culture-based techniques still necessary in many cases
Detection of pathogens even when antimicrobial treatment has been initiated	Initial cost to buy the equipment
Potential for better antibiotic utilisation and positive impact on: -nosocomial pneumonia management, shortening hospital stay and decreasing healthcare costs, -antibiotic stewardship programs	Not widely available among different institutions yet
Early identification of MDR pathogens should facilitate enhanced infection control practices and reduce spread	Further validation versus traditional diagnostic techniques needed and determination of the effect on antimicrobial prescribing, patient outcomes and resistance is needed

**Table 3:** Dosage and treatment duration of novel antibiotics for nosocomial pneumonia; other approved indications.

	NP (HAP and/or VAP): Dosage and Treatment Duration for NP	Other Approved Indications
Ceftolozane/tazobactam	HAP and VAP ^1^ Dosage: 3 g (2/1) every 8 h (h), 1-h IV infusion, *(Note: double dose compared to other indications)* Duration: 8–14 days (d)	cIAIs cUTIs (including acute pyelonephritis)
Ceftazidime/avibactam	HAP and VAP, including bacteraemic cases (bacteraemia associated with or suspected to be associated with HAP/VAP) ^1^ Dosage: 2.5 g (2/0.5) every 8 h, 2-h IV infusion Duration: 7–14 d	cIAI (in combination with metronidazole), cUTI (including pyelonephritis), Bacteraemia associated with or suspected to be associated with cIAI or cUTI Infections due to aerobic Gram-negative organisms in patients with limited treatment options
Meropenem/vaborbactam	HAP and VAP, including bacteraemic cases (bacteraemia associated with or suspected to be associated with HAP/VAP) ^1^ Dosage: 4 g (2/2) every 8 h, 3-h IV infusion Duration: 7–14 d	cIAI cUTI (including pyelonephritis), Bacteraemia associated with or suspected to be associated with cIAI or cUTI Infections due to aerobic Gram-negative organisms in patients with limited treatment option
Ceftobiprol medocaril	HAP (not for VAP) ^1^ Dosage: 500 mg every 8 h, 2-h IV infusion Duration: 7–14 d	CAP
Telavancin	HAP and VAP caused by *S. aureus* including bacteraemic cases (when no alternative treatment available) ^2^ Dosage: 10 mg/kg every 24 h, 1-h IV infusion Duration: 7–21 d	cSSSI caused by *S. aureus* including bacteraemic cases (when no alternative treatment available)

^1^ Need for dose adjustment in renal impairment (eGFR < 50 mL/min; decrease of dose, no change of intervals); no need for dose adjustment in liver impairment. ^2^ Need for dose adjustment in renal impairment (eGFR 30–50 mL/min; decrease of dose, no change of intervals)—not indicated for < eGFR 30 mL/min; liver impairment: caution in case of severe impairment (Child-Pugh C). IV: intravenous; cIAI: complicate intra-abdominal infection; cUTI: complicated urinary tract infection; cSSSI: complicated skin and skin structures infection.

**Table 4:** Spectrum of activity of novel antibiotics for the treatment of nosocomial pneumonia (HAP and/or VAP).

	ESBL	AmpC	KPC	OXA	MBL	Carb-R A.B.	MRSA
Ceftolozane/tazobactam ^1^	+	+/−	−	−	−	−	−
Ceftazidime/avibactam ^2^	++	+	+	+	−	−	−
Meropenem/vaborbactam ^3^	+	+	+	−	−	−	−
Ceftobiprol medocaril	−	−	−	−	−	−	+
Telavancin	−	−	−	−	−	−	+

NOTE: None of these novel antibiotics is active against VRE; ESBL: extended-spectrum beta-lactamases, KPC: *Klebsiella pneumoniae* carbapenemase, OXA: oxacillinase (refers to OXA carbapenemases), MBL: metallo-beta-lactamases, A.B.: *Acinetobacter baumannii*, Carb-R: carbapenem-resistant; ++: very active, +: active, −: not active. ^1^ Active against XDR-*P. aeruginosa*, Enterobacteriaceae (including some ESBL and AmpC producers); ^2^ Active against Enterobacteriaceae (including ESBL, AmpC, KPC and OXA-48 producers), MDR-*P. aeruginosa*; ^3^ Active against Enterobacteriaceae (including KPC, ESBL and AmpC producers, and carbapenem-resistant Enterobacteriaceae)—inactive against MDR *P. aeruginosa* (including carbapenem resistant strains).

## Data Availability

Not applicable.
